# *Listeria monocytogenes* Prevalence and Characteristics in Retail Raw Foods in China

**DOI:** 10.1371/journal.pone.0136682

**Published:** 2015-08-28

**Authors:** Shi Wu, Qingping Wu, Jumei Zhang, Moutong Chen, Ze′an Yan, Huijuan Hu

**Affiliations:** 1 School of Bioscience and Bioengineering, South China University of Technology, Guangzhou, PR China; 2 Guangdong Institute of Microbiology, State Key Laboratory of Applied Microbiology Southern China, Guangdong Provincial Key Laboratory of Microbial Culture Collection and Application, Guangdong Open Laboratory of Applied Microbiology, Guangzhou, PR China; USDA-ARS-ERRC, UNITED STATES

## Abstract

The prevalence and levels of *Listeria monocytogenes* in retail raw foods covering most provincial capitals in China were studied with testing of 1036 samples of vegetables, edible mushrooms, raw meat, aquatic products and quick-frozen products from September 2012 to January 2014. The total prevalence of *Listeria monocytogenes* was 20.0% (207/1036), and the most probable number (MPN) values of 65.7% of the positive samples ranged from 0.3 to 110 MPN/g. Geographical differences were observed in this survey, and the results of both qualitative and quantitative methods indicated that the levels in the samples from North China were higher than those in the samples from South China. A total of 248 isolates were analyzed, of which approximately half belonged to molecular serogroup 1/2a-3a (45.2%), followed by 1/2b-3b-7 (30.6%), 1/2c-3c (16.1%), 4b-4d-4e (5.2%) and 4a-4c (2.8%). Most of the isolates carried *hly* (100%), *inlB* (98.8%), *inlA* (99.6%), *inlC* (98.0%) and *inlJ* (99.2%), and 44.8% of the isolates were *llsX*-positive. Seventeen epidemic clones (ECs) were detected, with 7 strains belonging to ECI (2.8%) and 10 belonging to ECIII (4.03%). Resistance to clindamycin (46.8%) was commonly observed, and 59 strains (23.8%) were susceptible to all 14 tested antibiotics, whereas 84 (33.9%) showed an intermediate level of resistance or were resistant to two or more antibiotics, including 7 multi-resistant strains that exhibited resistance to more than 10 antibiotics. The data obtained in the present study provides useful information for assessment of the possible risk posed to Chinese consumers, and this information will have a significant public health impact in China. Furthermore, the presence of virulence markers, epidemic clones, as well as the antibiotic resistance amongst the isolates strongly implies that many of these strains might be capable of causing listeriosis, and more accurate treatment of human listeriosis with effective antibiotics should be considered. This research represents a more full-scale and systematical investigation of the prevalence of *L*. *monocytogenes* in retail raw foods in China, and it provides baseline information for Chinese regulatory authorities that will aid in the formulation of a regulatory framework for controlling *L*. *monocytogenes* with the aim of improving the microbiological safety of raw foods.

## Introduction

*Listeria monocytogenes* is a facultative intracellular pathogen that causes listeriosis, particularly in young, old, pregnant and immune-compromised individuals [1]. Infections with this bacterium are currently associated with a fatality rate of approximately 17%, which is the highest rate observed among foodborne pathogens [2]. *L*. *monocytogenes* is ubiquitous in the environment and can survive even under low temperatures and pH, high concentrations of salt or bile, oxidative stress, carbon starvation, and other adverse conditions [3]. Although ready-to-eat foods are important sources of *L*. *monocytogenes*, the contamination may occur in almost all types of raw food [4], with vegetables, milk, meat and seafood most frequently implicated [5]. Because ampicillin/penicillin and gentamicin are the primary antibiotics for listeriosis therapy, resistance to these antibiotics has been the focus of many previous studies [6–8]. In addition, single or multiple antibiotic-resistant *L*. *monocytogenes* strains isolated from food, the environment and clinics have also been frequently reported [9–11]. Consequently, it is necessary to increase the available data regarding the prevalence and antibiotic susceptibility of *L*. *monocytogenes* from various sources in different areas.

Although 13 serotypes of *L*. *monocytogenes* strains have been observed, serotypes 1/2a, 1/2b, 1/2c and 4b account for more than 95% of human listeriosis cases [12]. The results of multiplex polymerase chain reaction (PCR) indicated that these major serovars could be separated into distinct groups: I.1 (1/2a-3a), I.2 (1/2c-3c), II.1 (4b-4d-4e), II.2 (1/2b-3b-7) and III (4a-4c) [13]. According to Kathariou et al. [14], most major outbreaks result from a small number of epidemic clones (ECs) of *L*. *monocytogenes*, and Sauders et al. [15] suggested that many concurrent sporadic listeriosis cases were also caused by major ECs of *L*. *monocytogenes*. These ECs have been classified into seven groups (ECI, ECII, ECIII, ECIV, ECV, ECVI, and ECVII). ECI, ECII and ECIV within serotype 4b have been observed in several major outbreaks in different countries [14], whereas ECIII isolates within serotype 1/2a were identified during outbreaks in 1989 and 2000 in the United States and have been considered epidemiologically related because these isolates were detected in the same food processing plant with identical pulsed field gel electrophoresis (PFGE) patterns using different restriction enzymes [16]. Moreover, multi-virulence-locus sequence typing (MVLST) was used to accurately identify other three ECs: ECV within serotype 1/2a from human listeriosis cases in Canada over two decades [17]; ECVI within serotype 1/2b and ECVII within serotype 1/2a from the 2011 US listeriosis outbreak linked to cantaloupe [18]. Based on these findings, it is reasonable to speculate that previously identified ECs might be involved in future listeriosis cases and outbreaks. Therefore, identification and tracking of these ECs is important for understanding long-term transmission of *L*. *monocytogenes* and the establishment of efficient surveillance systems for this pathogen.

As an important foodborne pathogen, *L*. *monocytogenes* encompasses a spectrum of strains with varying virulence and pathogenicity. Differentiation between virulent and non-virulent strains is significant for evaluating the potential implications of the presence of this microorganism for food safety and public health [19]. Many putative virulence markers in *L*. *monocytogenes*, such as surface-associated internalins, have been implicated in the pathogenesis of human listeriosis [8]. The virulence factors InlA (which is responsible for the internalization of *L*. *monocytogenes* into epithelial intestinal cells), InlC (which plays an essential role in cell-to-cell spreading), and InlJ (which is directly involved in passage through the intestinal barrier and in subsequent stages of infection) are considered key virulence factors of *L*. *monocytogenes* [20]. In addition, the *llsX* gene produces another virulence factor, listeriolysin S (LLS), which plays a role in the survival of *L*. *monocytogenes* in polymorphonuclear neutrophils, contributes to the virulence observed in a mouse model, and might be important in the pathogenesis of human listeriosis [21].

The proportion of ready-to-eat foods that were found to be contaminated with *L*. *monocytogenes* in previous studies was high (> 6%) [22, 23], suggesting that the source materials (e.g., vegetables, meat or seafood) might have been the source of contamination. However, limited published nationwide data that can be used for qualitative and quantitative examination of this bacterium in China are available. Therefore, the aim of the present study was to investigate the prevalence and levels of *L*. *monocytogenes* isolates in retail raw foods from South China to North China and characterize these *L*. *monocytogenes* isolates according to their serogroups, antibiotic susceptibility profiles, and virulence potential by testing for the presence of significant virulence markers.

## Materials and Methods

### Sampling

**Fig 1:**
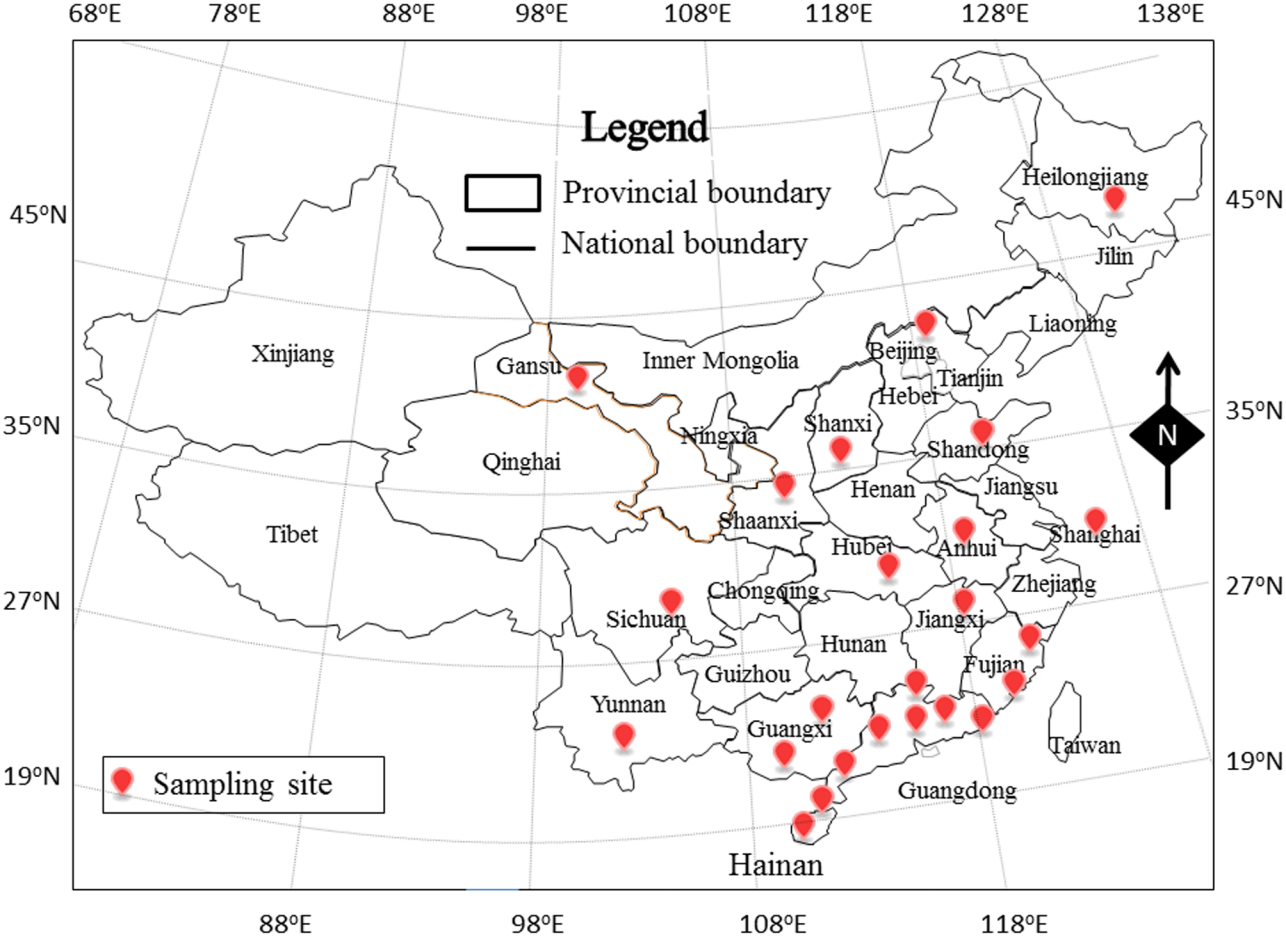
The locations of the sampling sites for this study in China. Based on 30° northern latitude, there are six cities in north China and 18 cities in South China.

From September 2012 until January 2014, 1036 retail raw food samples were collected from supermarkets, fairs and farmers’ markets (n = 134) in 24 cities in 14 provinces and two directly controlled municipalities in China (Fig 1). The samples, including vegetables, edible mushrooms (*Flammulina velutipes*, *Lentinus edodes*, *Volvariella volvacea*, *Auricularia auricula*, *Hypsizygus marmoreus*, and *Pleurotus eryngii*), meat and meat products (bacon/sausage, poultry, pork, mutton and beef), aquatic products (freshwater fish, shrimp and seafood) and quick-frozen products (frozen dumplings/steamed stuffed buns and frozen meat) (S1 Table), were placed in a cold box at a temperature of lower than 4°C, tightly sealed with sterile plastic wrap, and transported to an accredited laboratory and subjected to microbiological analysis within 24 h.

### Isolation and detection

The samples were qualitatively and quantitatively analyzed to determine the presence of *L*. *monocytogenes* (S1 Fig). For qualitative analysis, *L*. *monocytogenes* were isolated according to GB 4789.30–2010 for food microbiological examination of *L*. *monocytogenes* (National Food Safety Standards of China) with slight modifications. Briefly, 25 g of the food sample was homogenized in 225 mL of *Listeria* enrichment broth I culture (LB_1_, Huankai, Guangzhou, China), followed by incubation at 30°C for 24 h. Subsequently, a 0.1-mL sub-sample of LB_1_ enrichment broth culture was added to 10 mL of *Listeria* enrichment broth II culture (LB_2_, Huankai, Guangzhou, China) and incubated at 30°C for an additional 24 h. The most probable number (MPN) method described by Gombas et al. [24] was adapted for use in this study for quantitative analysis. The same samples were incubated in half Fraser broth (FB_1_, Huankai, Guangzhou, China) without supplementation at 20°C for 1 h. Subsequently, 10-mL, 1-mL and 0.1-mL aliquots were transferred to tubes containing 0 mL, 10 mL and 10 mL full Fraser broth (FB_2_, Huankai, Guangzhou, China) at 30°C for 48 h for quantitative analysis.

A loopful of enrichment broth culture (10 μL) (LB_2_, FB_1_ and FB_2_ broth cultures) was streaked onto *Listeria*-selective plates (CHROM-agar, Paris, France) and incubated at 37°C for 24 h. One to four colonies with blue halos were purified on tryptic soy agar with yeast extract (TSA-YE). The purified colonies were analyzed via Gram staining, catalase tests and oxidase tests, and the Micro ID *Listeria* identification system was used (Microgen, Camberley, UK).

### Molecular serogroup identification

All isolates were analyzed by multiplex PCR [13] using five primers (*lmo*0737, *lmo*1118, ORF2819, ORF2110 and *prs*). This method was used to place the *L*. *monocytogenes* strains into five serovar groupings: I.1 (1/2a-3a), I.2 (1/2c-3c), II.1 (1/2b-3b-7), II.2 (4b-4d-4e) and III (4a-4c).

### Detection of virulence markers

After confirming the *L*. *monocytogenes* species, the isolates were also identified using duplex PCR detection of the *hly* (707 bp) and *inlB* (367 bp) genes, which are specific to *L*. *monocytogenes*, as previously described [25]. Multiplex PCR was also used to identify the virulence genes *inlA*, *inlC* and *inlJ* [26]. The *llsX* gene was detected through PCR assays to identify LLS-positive *L*. *monocytogenes* strains [27]. Presumptive major ECs (ECI, ECII and ECIII) were identified in the isolates as previously described [28]. The primers and PCR conditions are presented in Table 1.

**Table 1:** Primer sequences, PCR preparations and PCR conditions used for *L*. *monocytogenes* isolates in this study.

PCR test	Target gene	Product size (bp)	Primer sequences (5’-3’)	PCR preparation	PCR conditions	Reference
dPCR1	***inlB*, *hly***	367, 707	inlB-F: GATATTGTGCCACTTTCAGGTT, inlB-R: CCTCTTTCAGTGGTTGGGTT; hly-F: GTTAATGAACCTACAAGACCTTCC, hly-R: ACCGTTCTCCACCATTCCCA	10 μl 2× DreamTaq mastermix, 7 μl nuclease-free water, 80 ng template DNA, and 0.06 μM each primer	5 min at 94°C, 35 cycles of 94°C for 35 s, 60°C for 45 s, 72°C for 1 min and a final extension at 72°C for 10 min	Xu et al. (2009)
mPCR2	***inlA***, ***inlC***, ***inlJ***	800, 517, 238	inlA-F: ACGAGTAACGGGACAAATGC, inlA-R: CCCGACAGTGGTGCTAGATT; inlC-F: AATTCCCACAGGACACAACC, inlC-R: CGGGAATGCAATTTTTCACTA; inlJ-F: TGTAACCCCGCTTACACACAGTT, inlJ-R: AGCGGCTTGGCAGTCTAATA	12.5 μl 2× DreamTaq mastermix, 6 μl nuclease-free water, 80 ng template DNA, 0.4 μM *inlA* primer, 0.3 μM *inlC* primer, and 0.2 μM *inlJ* primer	2 min at 94°C, 30 cycles of 94°C for 30 s, 55°C for 30 s, 72°C for 1 min and a final extension at 72°C for 10 min	Liu et al. (2007)
PCR3	***llsX***	200	llsX-F: TTATTGCATCAATTGTTCTAGGG, llsX-R: CCCCTATAAACATCATGCTAGTG	12.5 μl 2× DreamTaq mastermix, 3.5 μl nuclease-free water, 80 ng template DNA, and 0.4 μM primer	3 min at 95°C, 45 cycles of 95°C for 30 s, 60°C for 1 min, 72°C for 1 min and a final extension at 72°C for 10 min	Clayton et al., 2011
mPCR4	**ECI**, **ECII**, **ECIII**	303, 889, 497	ECI-F: AATAGAAATAAGCGGAAGTGT, ECI-R: TTATTTCCTGTCGGCTTAG; ECII-F: ATTATGCCAAGTGGTTACGGA, ECII-R: ATCTGTTTGCGAGACCGTGTC; ECIII-F: TTGCTAATTCTGATGCGTTGG, ECIII-R: GCGCTAGGGAATAGTAAAGG	12.5 μl 2× DreamTaq mastermix, 6 μl nuclease free water, 80 ng template DNA, 0.4 μM ECI primer, 0.7 μM ECII primer, and 0.3 μM ECIII primer	3 min at 95°C prior to 15 cycles of 1 min at 94°C, 1 min with a touchdown from 55°C to 51°C (3 cycles per temperature), and 1 min at 72°C, followed by 15 cycles of 1 min at 94°C, 1 min at 50°C, and 1 min at 72°C with 1 final cycle for 8 min at 72°C	Chen & Knabel (2007)

### Antibiotic susceptibility tests

Antibiotic susceptibility tests of *L*. *monocytogenes* isolates were performed via standard disk diffusion on Mueller—Hinton agar incubated at 37°C for 24 h using the Kirby—Bauer method [29]. The following 14 antibiotics (Oxoid, UK) were classified into 10 different groups according to the WHO [30]: gentamicin (CN, 10 μg), streptomycin (S, 25 μg), kanamycin (K, 30 μg), chloramphenicol (C, 30 μg), rifampicin (RD, 5 μg), cephalothin (KF, 30 μg), vancomycin (VA, 30 μg), clindamycin (DA, 2 μg), erythromycin (E, 15 μg), ampicillin (AMP, 10 μg), mezlocillin (MEZ, 30 μg), penicillin G (P, 5 U), ciprofloxacin (CIP, 5 μg) and tetracycline (TE, 30 μg). *Staphylococcus aureus* ATCC29213 and *Escherichia coli* ATCC25922 were used as quality control organisms [31].

### Statistical analysis

The bacterial numbers were converted to base-10 logarithms for statistical analysis. MPN values < 0.3 MPN/g were set to 0.15, and MPN values > 110 MPN/g were assigned the maximum value for this test [32]. The chi-square test was used to determine differences in the prevalence and levels of *L*. *monocytogenes-*positive samples between qualitative variables. All statistical analyses were performed using the SPSS v21.0 software package.

## Results and Discussion

### Prevalence and levels of *L*. *monocytogenes* in retail raw foods

A total of 1036 raw food samples were collected from supermarkets, fairs and farmers’ markets (n = 134) in 24 Chinese cities. Overall, the average prevalence of *L*. *monocytogenes* was 20.0% (207/1036), and the MPN values of 65.7% of the positive samples ranged from 0.3 to 110 MPN/g. The geometric mean was 18.24 MPN/g. Out of the 207 positive samples, 23 (11.1%) had values that exceeded 100 MPN/g, whereas 132 (63.8%) were below 1 MPN/g. Nowadays, there is no standard limit of *L*. *monocytogenes* in Chinese retail raw food. Although the US has proposed a zero-tolerance policy for *L*. *monocytogenes* in food, the European Union (EU) has proposed a limit of 100 cfu/g; the highest fraction of samples with values that exceeded this limit was observed at the end of the foods’ shelf-life [33]. Thus, on the whole, levels of *L*. *monocytogenes* in raw foods in China were not very excessive.

The distribution of *L*. *monocytogenes* among the different sampling sites is presented in Table 2.

**Table 2:** Prevalence and level of *Listeria monocytogenes* at different sampling sites.

Sampling site		Sampling time (year.month)	No. of samples	No. (%) of positive samples	No. of positive samples by quantitative methods (MPN/g)				*L*. *monocytogenes* level (MPN/g)^b^
City	Province				< 1	≥1–< 10	≥10–< 100	≥ 100	
Lanzhou	Gansu	2012.11	37	8 (21.6)	4	2	1	1	20.14
Haerbin	Heilongjiang	2012.11	37	14 (37.8)	11	1	0	2	16.61
Xi’an	Shaanxi	2012.12	37	9 (24.3)	6	2	0	1	13.18
Taiyuan	Shanxi	2012.12	37	11 (29.7)	4	2	3	2	26.75
Beijing^a^	--	2013.01	37	13 (35.1)	5	1	2	5	46.07
Jinan	Shandong	2013.01	37	13 (35.1)	6	6	1	0	4.71
**North China**	**--**		**222**	**68 (30.6)**	**36**	**14**	**7**	**11**	**21.24**
Shanghai^a^	--	2012.09	37	9 (24.3)	4	4	0	1	13.83
Hefei	Anhui	2012.09	37	10 (27.0)	6	2	0	2	23.32
Nanchang	Jiangxi	2012.09	37	3 (8.1)	1	1	1	0	16.1
Wuhan	Hubei	2012.10	37	3 (8.1)	2	1	0	0	1.58
Chengdu	Sichuan	2012.10	37	5 (13.5)	3	2	0	0	2.99
Kunming	Yunnan	2012.10	37	6 (16.2)	5	1	0	0	1.71
Guangzhou	Guangdong	2013.02~2013.04	185	44 (23.8)	30	5	5	4	13.61
Shenzhen	Guangdong	2013.05	37	4 (10.8)	3	1	0	0	0.88
Shaoguan	Guangdong	2013.05	37	5 (13.5)	4	1	0	0	0.58
Zhanjiang	Guangdong	2013.05	37	13 (35.1)	9	2	1	1	10.92
Shantou	Guangdong	2013.06	37	8 (21.6)	6	1	0	1	14.34
Heyuan	Guangdong	2013.06	37	2 (5.4)	1	1	0	0	1.95
Haikou	Hainan	2013.12	37	4 (10.8)	2	0	0	2	55.11
Sanya	Hainan	2014.01	37	3 (8.1)	3	0	0	0	0.41
Beihai	Guangxi	2013.12	37	5 (13.5)	3	1	0	1	22.76
Nanning	Guangxi	2013.11	37	3 (8.1)	3	0	0	0	0.22
Fuzhou	Fujian	2013.11	37	4 (10.8)	4	0	0	0	0.3
Xiamen	Fujian	2013.11	37	8 (21.6)	7	0	1	0	2.27
**South China**	**--**		**814**	**139 (17.1)**	**96**	**23**	**8**	**12**	**10.16**

^a^ These two cities are direct-controlled municipalities.

^b^ The values are weighted averages shown as the geometric means of positive samples.

Among the 24 cities, the prevalence of *L*. *monocytogenes* varied in different regions, ranging from 5.4% in Heyuan to 37.8% in Haerbin. Moreover, the developed cities, such as Beijing (35.1%), Shanghai (24.3%) and Guangzhou (23.8%) showed higher prevalence. In total, twelve of the 24 cities (50%) contained *L*. *monocytogenes*-positive samples, with MPN values of above 100 MPN/g. The most severe contamination level among the 24 cities was observed in Haikou (55.11 MPN/g), followed by Beijing (46.07 MPN/g), Taiyuan (26.75 MPN/g), Hefei (23.32 MPN/g) and Lanzhou (20.14 MPN/g). A total of 222 samples were collected from North China (Lanzhou, Haerbin, Xi’an, Taiyuan and Beijing). The average prevalence of *L*. *monocytogenes* in these samples was 30.6%, with a mean level of 21.24 MPN/g. The prevalence of *L*. *monocytogenes* in Lanzhou was 21.6% (8/37), and it was 37.8% (14/37) in Haerbin, 24.3% (9/37) in Xi’an, 29.7% (11/37) in Taiyuan, and 35.1% (13/37) in Beijing. Based on the MPN value, 11 positive samples (11/68, 16.2%) had a *L*. *monocytogenes* density that exceeded 100 MPN/g, and 36 positive samples (36/68, 52.9%) had a *L*. *monocytogenes* density of below 1 MPN/g. In South China, *L*. *monocytogenes* was detected in 139 of the 814 samples (17.1%); of these samples, 12 (8.6%) had an MPN value that exceeded 100 MPN/g, and 96 (69.1%) had a value of less than 1 MPN/g. Based on this result, the prevalence of this bacterium in raw foods from North China was significantly higher than that in foods from South China (p < 0.001, χ^2^ test); however, the differences in the MPN values were not significant (p > 0.05, χ^2^ test). This difference in prevalence was especially obvious for *L*. *monocytogenes* in fresh meat, which showed 51.4% of prevalence (19/37) in North China whereas 16.2% (22/136) in South China (data not shown). This geographic difference may be partially attributed to the psychrotolerant ability of *L*. *monocytogenes* [34]. Due to the high-low latitude climate links (Fig 1), the climate in North China is remarkably colder than that in South China giving *L*. *monocytogenes* more opportunity to multiply than other foodborne pathogens. Therefore, local retailers, wholesalers and relevant regulators should pay more attention to *L*. *monocytogenes* contamination of retail food.

Table 3 lists the analyzed food products classified into five categories, as well as the prevalence and level of *L*. *monocytogenes* in these foods.

**Table 3:** Prevalence and level of *Listeria monocytogenes* in different retail raw foods.

Product	No. of samples	No. (%) of positive samples	No. of positive samples determined by quantitative methods in the MPN/g range					*L*. *monocytogenes* level (MPN/g)^b^
			< 1	≥1–< 10	≥10–< 100		≥ 100	
**Meat and meat products**	**196**	**41 (20.9)**	**25**	**11**	**2**		**3**	**10.23**
Pork	87	24 (27.6)	13	8	1	2		11.69
Beef	23	8 (34.8)	6	2	-	-		0.99
Poultry	57	7 (12.3)	5	1	1	-		2.87
Mutton	6	2 (33.3)	1	-	-	1		55.37
Cured meat/sausage	23	0 (0.0)	-	-	-	-		-
**Aquatic products**	**280**	**18 (6.4)**	**15**	**0**	**1**	**2**		**13.73**
Seafood	84	7 (8.3)	6	-	-	1		15.89
Freshwater fish	145	10 (6.9)	8	-	1	1		13.58
Shrimp	51	1 (2.0)	1	-	-	-		0.15
**Quick-frozen food** ^a^	**196**	**88 (44.9)**	**66**	**16**	**3**	**3**		**5.4**
Dumplings/steamed stuffed buns	84	27 (32.1)	25	2	-	-		0.46
Frozen meat	112	61 (54.5)	41	14	3	3		7.58
**Edible mushrooms**	**224**	**52 (23.2)**	**21**	**9**	**9**	**13**		**33.67**
*Flammulina velutipes*	81	45 (55.6)	16	8	9	12		35.45
Other edible mushrooms	143	7 (4.9)	5	1	-	1		20.03
**Vegetables**	**140**	**8 (5.7)**	**5**	**1**	**0**	**2**		**28.15**
**Total**	**1036**	**207 (20.0)**	**132**	**37**	**15**	**23**		**18.24**

^a^ All quick-frozen foods were stored at -10°C before being sold.

^b^ The values are the weighted averages, shown as geometric means of the positive samples.

Among the analyzed categories, quick-frozen food was the most frequently contaminated with *Listeria monocytogenes*, with a prevalence that reached 44.9%. The prevalence of *L*. *monocytogenes* in dumplings/steamed stuffed bun was 32.1% (27/84), and that in frozen meats was 54.5% (61/112). These values are remarkably higher than those reported in previous studies [35, 36]. In contrast, the mean level of *L*. *monocytogenes* in quick-frozen products was the lowest in this survey (5.40 MPN/g). As we know, freezing is an excellent way to preserve food quality and to minimize the incidence of foodborne pathogens; however, *L*. *monocytogenes* grows at refrigeration temperatures and can also survive during frozen storage [37]. In fact, Simpson et al. [38] suggested that freezing had little effect on *L*. *monocytogenes* in food preserved at -15°C, regardless of the product formulation. Only when a large amount of bacteria was present (3.9 log CFU/cm^2^) did freezing result in noticeable (≤1 log CFU/cm^2^) but not significant (p ≥ 0.05) reductions. Therefore, although it is unclear whether these quick-frozen products acquire *L*. *monocytogenes* during production or whether these foods are primary reservoirs for this bacterium, there is an urgent need for implementing hygienic standards in food cold-chain management and food processing.

The overall prevalence of *L*. *monocytogenes* isolates in raw meat (23.7%, 41/173) was higher than that reported in previous studies in China, demonstrating that *L*. *monocytogenes* contamination has increased over the years, with a prevalence of 7.1% in 2000 to 2007, 11.67% in 2006 to 2009, 12% in 2008 to 2009, and 23.7% in 2012 to 2014 (this study) [39–44]. Moreover, 7.3% of *L*. *monocytogenes*-positive raw meats had MPN values of at least 100 MPN/g. This trend may be related to the rapid development of the meat processing industry in China because post-slaughter processing is a significant cause of meat contamination, and the level of contamination is amplified in the chilling and cutting room environment [45].

Aquatic products, particularly seafood, have high nutritional value, and consumption of these products has increased. Among the 280 aquatic products examined in the survey, *L*. *monocytogenes* was the most often encountered in seafood (8.3%, 7/84), followed by freshwater fish (6.9%, 10/145) and shrimp (2.0%, 1/51); the MPN values were less than 100 MPN/g in most of the samples (16/18). In China, these values were higher than those previously reported by Chen et al. [40] (a prevalence of 2.7% from 2000 to 2007) but lower than those obtained by Wang et al. [43], who reported that the prevalence of *L*. *monocytogenes* in seafood reached 13.06% from 2008 to 2009. In other countries, the prevalence ranged from 1.4% to 39.0% [46–50]. Because of these differences, the prevalence of *L*. *monocytogenes* in aquatic products has been associated with the sample sizes, sample types and geographic locations, which might impact the presence of these organisms [50]. For instance, Gram [51] reviewed studies that reported large variations, with 1% to 34% of the raw fish samples that entered fish processing plants testing positive for *L*. *monocytogenes*. Still, seafood frequently triggers regulatory alerts in importing countries. Contamination with *L*. *monocytogenes* has led to product recalls, resulting in direct and indirect financial losses [52]. Therefore, *L*. *monocytogenes* contamination significantly impacts the seafood trade and should receive increased industry attention.

*L*. *monocytogenes* was frequently detected in edible mushrooms (23.2%, 52/224), particularly in *Flammulina velutipes*, for which the fraction of samples that were contaminated was 55.6%. The percentages of *Flammulina velutipes* and other edible mushrooms that tested positive for *L*. *monocytogenes* significantly differed (p < 0.001, χ^2^ text). Based on this anomaly, we further investigated mushroom production plants to determine the potential source of *L*. *monocytogenes* contamination and observed that the contamination originated from mycelium-scraping machinery, which could potentially contaminate both the products and the upstream packaging equipment [53]. Interestingly, in both the mushroom production plant survey and this nationwide retail products survey, the prevalence of *L*. *monocytogenes* in *Flammulina velutipes* was always high. Thus, a correlation between *Flammulina velutipes* and *L*. *monocytogenes* might exist; however, further research is needed to determine the reason for this correlation. Furthermore, although vegetables had a low contamination frequency (5.7%, 8/140), two positive samples (one tomato sample and one lettuce sample) had MPN levels exceeding 100 MPN/g based on quantitative analysis. Because these two types of samples are always consumed raw, more consumers may be exposed to *L*. *monocytogenes*, and precautions should be taken to prevent this exposure.

Listeriosis is almost always caused by exposure to a food source that was contaminated somewhere along the food chain, and raw materials that enter processing facilities carrying both transient and persistent strains are major sources of contamination [54]. Some strains have been shown to persist for months or even years [12]. Although most of raw food is normally eaten after cooking, the risk of listeriosis should not be underestimated, as the consumption of raw foods such as carpaccio, sushi, and undercooked hamburger is increasing worldwide. On the other hand, it may be necessary to improve hygiene and provide adequate storage conditions, both at the food production level and in retail establishments, to avoid a high level of growth of this pathogen because cross-contamination is a major factor for the introduction of *L*. *monocytogenes* to foods. The nationwide data regarding the qualitative and quantitative prevalence and level of *L*. *monocytogenes* isolated from retail raw food in China were supplemented based on the results of wide-scale and systematic investigation. These data, in addition to data from studies carried out in other countries, should help to establish local and international standards for levels of *L*. *monocytogenes* in foods.

### Molecular serogroups of *L*. *monocytogenes* isolates

A total of 248 *L*. *monocytogenes* isolates were collected from the retail raw foods, comprising 49 isolates from raw meat, 20 isolates from aquatic products, 108 isolates from quick-frozen food, 61 isolates from edible mushrooms and 10 isolates from vegetables. The serogroups carried by the isolates are presented in Table 4.

**Table 4:** Serogroups of *Listeria monocytogenes* strains in positive samples.

Source	No. (%) of serotype-positive isolates					Total food isolates
	1/2a-3a	1/2c-3c	4b-4d-4e	1/2b-3b-7	4a-4c	
**Raw meat**	23 (46.9)	14 (28.6)	3 (6.1)	7 (14.3)	2 (4.1)	49
**Aquatic products**	4 (20)	2 (10)	3 (15)	8 (40)	3 (15)	20
**Quick-frozen food**	50 (46.3)	22 (20.4)	7 (6.5)	28 (25.9)	1 (0.9)	108
**Edible mushrooms**	32 (52.5)	0	0	28 (45.9)	1 (1.6)	61
**Vegetables**	3 (30)	2 (20)	0	5 (50)	0	10
**Total**	112 (45.2)	40 (16.1)	13 (5.2)	76 (30.6)	7 (2.8)	248

Serotypes 3a, 3c, 4d, 4e, 3b, 7 and 4c are rarely (<5%) observed in foods and clinics samples, and in the current study, serotypes 1/2a (112/248, 45.2%), 1/2b (76/248, 30.6%) and 1/2c (40/248, 16.1%) were the major types observed from foods, and 13 isolates (5.2%) and 7 isolates (2.8%) belonged to serotypes 4b and 4a, respectively, which is consistent with the findings of previous studies in other countries [20, 42]. The most prevalent serotype in this study, 1/2a (45.2%), showed good ecological fitness in foods and food-associated environments. Several authors have speculated that this serotype has an increased ability to persist in food and seems to carry more plasmids, which often confer resistance to toxic metals and other compounds [10].

Serotype 4b, which is associated with most major outbreaks of invasive forms of listeriosis [55], was observed only in animal-derived foods (raw meat, aquatic products and quick-frozen products). Although 5.2% of the strains belonged to this serotype, the overrepresentation of serotype 4b strains among human listeriosis outbreaks and the sporadic cases suggest that these strains are more virulent and might pose a greater health threat than the other serotype strains. However, the serogroups most often found associated with edible mushrooms and vegetables were 1/2a-3a and 1/2b-3b-7 (95.8%). The *L*. *monocytogenes* serotypes showed different distributions in different food types. This may be due to the fact that some serotypes of *L*. *monocytogenes* can outcompete other serotypes during growth environment as well as their niches, distributions and modes of transmission.

### Prevalence of virulence markers

All of the isolates harbored *hly*, and at least one of the internalin genes (*inlA*, *inlB*, *inlC* and *inlJ*) was present in a majority of the isolates (Table 5).

**Table 5:** Virulence markers of 248 *Listeria monocytogenes* strains in positive samples.

Virulence markers	No. (%) of positive samples	No. (%) of serotype-positive isolates				
		1/2a-3a	1/2c-3c	4b-4d-4e	1/2b-3b-7	4a-4c
***hly***	248/248 (100)	112 (100)	40 (100)	13 (100)	76 (100)	7 (100)
***inlB***	245/248 (98.8)	111 (99.1)	38 (95)	13 (100)	76 (100)	7 (100)
***inlA***	247/248 (99.6)	111(99.1)	40 (100)	13 (100)	76 (100)	7 (100)
***inlC***	243/248 (98.0)	111(99.1)	40 (100)	13 (100)	76 (100)	3 (42.9)
***inlJ***	246/248 (99.2)	110 (98.2	40 (100)	13 (100)	76 (100)	7 (100)
***llsX***	111/248 (44.8)	38 (33.9)	9 (22.5)	10 (76.9)	48 (63.2)	6 (85.7)
**ECI**	7/248 (2.8)	0 (0)	0(0)	7 (53.8)	0(0)	0(0)
**ECII**	0/248 (0)	0(0)	0(0)	0(0)	0(0)	0(0)
**ECIII**	10/248 (4.03)	10 (8.9)	0(0)	0(0)	0(0)	0(0)

Most of these isolates possessed virulence genes that were similar to those of clinical isolates [56]. Sant’Ana et al. [57] and Shen et al. [20] have also detected these virulence markers in *L*. *monocytogenes* from food samples, reporting similar results. In addition, a correlation between *inlC* and serotype 4a/4c was observed: 4 out of 5 *inlC*-negative isolates belonged to serotype 4a/4c, whereas the remaining isolate was an *inlA*-*inlC*-negative strain. This finding has also been previously observed [58]. den Bakker et al. [59] have demonstrated an association between the presence of *inlC* genes in pathogenic strains and the absence of these genes in non-pathogenic strains by analyzing the genomes of thirteen *Listeria* organisms representing six *Listeria* species.

The *llsX* gene (encoding LLS) was employed as a genetic marker to detect *L*. *monocytogenes* with *Listeria* pathogenicity island 3 (LIPI-3) [21], and isolates from the present study exhibited 44.8% (111/248) positivity. This rate is higher than those obtained by Dmowska et al. [60] and Shen et al. [20], who observed that 7.4% and 12.4% of *llsX*-positive samples from beef and retail meat, respectively. Although all strains isolated from raw meat and quick-frozen foods contained *inlA*, *inlC* and *inlJ* genes, most of these strains (34/49 and 83/108) lacked the *llsX* gene. Generally, this ferrous transport gene (siderophore) was present in lineage I isolates (including serotypes 1/2b and 4b) but was absent among lineage II isolates [61]. Half of the lineage I strains, including those involved in outbreaks, carried this gene. *L*. *monocytogenes* comprises multiple strains that exhibit varying virulence potential. Although the differences in the virulence potential is not completely due to the presence of these genes, positivity for these virulence makers plays an important role in the pathogenicity of *L*. *monocytogenes*. Thus, detection of these markers may represent a quick approach for preliminary discrimination of potentially virulent strains from avirulent strains of *L*. *monocytogenes* [26]. The high prevalence of these genes in isolates derived from food products indicates that they may not serve as sufficient indicators for pathogenic *L*. *monocytogenes* and that their sequences (e.g., premature stop codon mutations in *inlA*) may be further investigated to explore differences, which could help in differentiating pathogenic from less pathogenic strains.

However, the serotypes of worldwide *L*. *monocytogenes* included ECs that have been related to several major outbreaks in the US and Europe [14, 62]. These ECs, detected in 2.8% of the ECI isolates and 4.03% of the ECIII isolates in this study, still showed low percentage in food and food processing as described previously [20]. Nonetheless, EC is defined as isolates with a presumably common ancestor that are genetically related; thus, ECs facilitate strain characterization and the integration of subtype data into inspection programs that are based on assessment of relative risks [15]. *L*. *monocytogenes* EC strains possess unique adaptations that explain the frequent involvement of these bacteria in listeriosis outbreaks. Their presence in retail raw foods is a great threat to public health.

### Antibiotic susceptibility

*L*. *monocytogenes* is naturally sensitive to a wide spectrum of antibiotics; however, recently, a continuous pattern of emergence of resistant strains has been reported [7, 10, 63]. Fourteen antibiotics from 10 different groups were used in the present study to assess resistance of the *L*. *monocytogenes* isolates. The results (Table 6) revealed that penicillin G was the only antibiotic to which all 248 *L*. *monocytogenes* isolates were susceptible.

**Table 6:** Results of antimicrobial susceptibility tests of *Listeria monocytogenes* isolates obtained from retail raw food in China.

**Antimicrobial group**	Antibiotic	Antimicrobial class^a^ according to the WHO	No. (%) of *L*. *monocytogenes* (*n* = 248)		
			Susceptible	Intermediate	Resistant
Aminoglycosides	Gentamicin	CI	241 (97.2)	4 (1.6)	3 (1.2)
	Streptomycin	CI	234 (94.4)	2 (0.8)	12 (4.8)
	Kanamycin	HI	236 (95.2)	4 (1.6)	8 (3.2)
Amphenicols	Chloramphenicol	HI	228 (91.9)	13 (5.2)	7 (2.8)
Ansamycins	Rifampicin	CI	239 (96.4)	6 (2.4)	3 (1.2)
Cephalosporins	Cephalothin	HI	241 (97.2)	1 (0.4)	6 (2.4)
Glycopeptides	Vancomycin	CI	242 (97.6)	5 (2.0)	1 (0.4)
Lincosamides	Clindamycin	I	81 (32.7)	49 (19.8)	116 (46.8)
Macrolides	Erythromycin	CI	238 (96.0)	7 (2.8)	3 (1.2)
Penicillins	Ampicillin	CI	231 (93.1)	--	17 (6.9)
	Mezlocillin	CI	245 (98.8)	2 (0.8)	1 (0.4)
	Penicillin G	CI	248 (100)	--	0 (0)
Quinolones	Ciprofloxacin	CI	178 (71.8)	60 (24.2)	10 (4.0)
Tetracyclines	Tetracycline	HI	222 (89.5)	1 (0.4)	25 (10.1)

^a^ CI, critically important; HI, highly important; I, important

However, many isolates were resistant to clindamycin (116/248, 46.8%), and several isolates were resistant to tetracycline (25/248, 10.1%), ampicillin (17/248, 6.9%), streptomycin (12/248, 4.8%), ciprofloxacin (10/248, 4.0%), kanamycin (8/248, 3.2%), chloramphenicol (7/248, 2.8%), cephalothin (6/248, 2.4%), gentamicin (3/248, 1.2%), rifampicin (3/248, 1.2%) or erythromycin (3/248, 1.2%). Among the 248 isolates, only 59 strains (23.8%) were susceptible to all 14 tested antibiotics, whereas 84 strains (33.9%) showed an intermediate level of resistance or were resistant to 2 or more antibiotics. Moreover, we detected 7 strains that were resistant to more than 10 antibiotics, of which 4 isolates were collected from raw meat, 2 isolates were obtained from quick-frozen foods and one isolate was obtained from edible mushrooms. These findings confirmed that the prevalence of antibiotic resistance in *L*. *monocytogenes* may be increasing [23, 42].

The high prevalence of clindamycin-resistant *L*. *monocytogenes* isolates observed in the present study is consistent with that demonstrated in previous reports [8]. The effect of clindamycin on *Listeria* is controversial because contradictory results have been previously reported in a study describing the isolation of clindamycin-susceptible strains from raw meats, meat products and cheeses in Greece [64]. Clindamycin resistance might be associated with excessive use of this drug in veterinary and human medicine [65]. *L*. *monocytogenes* isolates were also resistant to tetracycline, ampicillin, streptomycin and ciprofloxacin. Considering that these antibiotics have been increasingly used as supplements in animal feed, as growth promoters and for the treatment of human disease [66], it is not surprising that resistant strains are more commonly observed. Furthermore, *L*. *monocytogenes* can transfer by conjugation of enterococcal and streptococcal plasmids, as well as transposons carrying antibiotic-resistance genes. from *Enterococcus-Streptococcus* to *Listeria* and between species of *Listeria* [67]. Additionally, mutations in chromosomal genes can play a role in conferring antibiotic resistance to *Listeria* species [65]. Therefore, a high percentage (33.9%) of multi-resistant isolates was found implying that new resistance genes may be acquired and transferred.

Godreuil et al. [68] have reported that antibiotic resistance likely results from active efflux associated with the overexpression of *lde* genes that encode a naturally occurring efflux pump. *L*. *monocytogenes* is exposed to a wide variety of drugs in nature; thus, causing adaptation of *Listeria* to environmental challenges through active efflux. However, attention should be focused on the high level of antimicrobial resistance (76.2%) of *L*. *monocytogenes* that was observed in the present study. The controlled use of antimicrobials would limit the emergence of drug-resistant bacteria.

## Conclusions

A systematic investigation of the prevalence and contamination levels of *L*. *monocytogenes* isolated from retail raw foods was performed. Most of the provincial capitals in China were included in this study, showing a full-scale geographic distribution. In various food categories, the potential risks of infection with *L*. *monocytogenes*, specifically for edible mushroom *Flammulina velutipes* (55.6% positivity for *L*. *monocytogenes*), should draw public attention. There was little variability among the molecular serogroup isolates in terms of their geographic distribution among China and other countries. Based on the presence of virulence markers, a subset of *L*. *monocytogenes* isolates could potentially cause human diseases. Furthermore, the discovery of many antibiotic-resistant isolates, which represent a public health problem, could be helpful for improving the treatment of listeriosis. This study is a full-scale, systematic investigation of the prevalence of *L*. *monocytogenes* in retail raw foods in China and the contamination of these foods, and it provides baseline information for Chinese regulatory authorities to allow the formulation of a regulatory framework for controlling *L*. *monocytogenes* and to improve the microbiological safety of raw foods.

## Supporting Information

S1 Fig Procedures for qualitative detection and quantitative analysis of *L*. *monocytogenes* in retail raw foods. (TIF)

S1 Table Information regarding the samples assessed in this study. (PDF)
