# Supplementary figures and images for: Listeria monocytogenes Prevalence and Characteristics in Retail Raw Foods in China
Source: PLoS One. 2015 Aug 28;10(8):e0136682. doi: 10.1371/journal.pone.0136682 (PMC4552630; doi:10.1371/journal.pone.0136682)

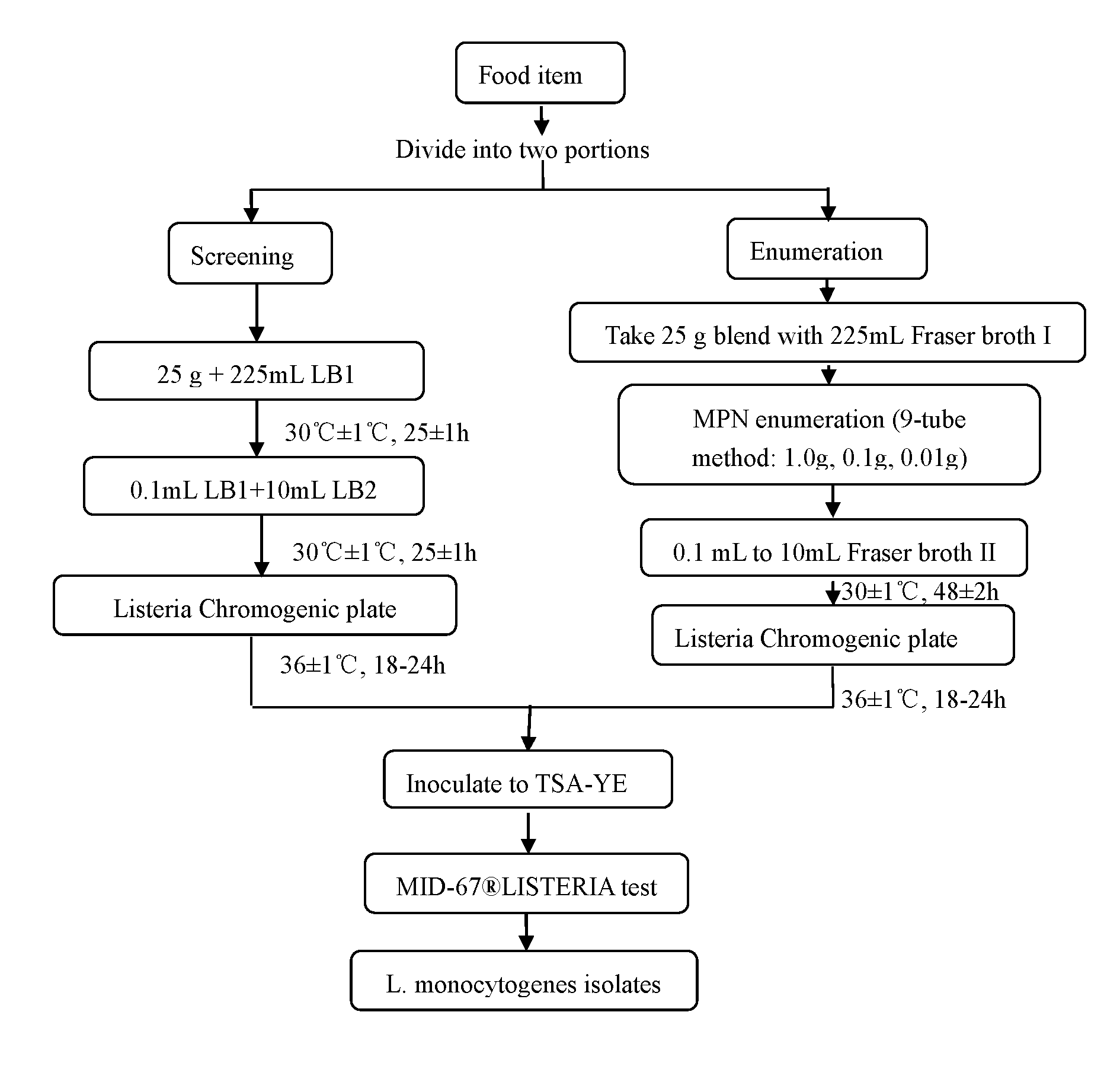

Supplement: S1 Fig — (TIF) [file pone.0136682.s001.tif]
