# Supplementary material for: Listeria monocytogenes Prevalence and Characteristics in Retail Raw Foods in China
Source: PLoS One. 2015 Aug 28;10(8):e0136682. doi: 10.1371/journal.pone.0136682 (PMC4552630; doi:10.1371/journal.pone.0136682)
Supplement: S1 Table — (PDF) [file pone.0136682.s002.pdf]

S1 Table The information of samples in this study

	Product type	NO. Sample	Name	Sample city	Source	Date in produced	Purchase Location	environment	sample character
1		SHC801	pork	Shanghai	Shanghai	20120905	Carrefour	box-packed in cold storage	fresh meat slices
2		SHC802	pork	Shanghai	Shandong	20120905	Carrefour	box-packed in cold storage	minced meat
3		SHC803	pork	Shanghai	Shanghai	20120905	Tesco	box-packed in cold storage	minced meat
4		SHJ822	Beef	Shanghai	Shanghai	20120905	Liuli farmer's market	cold storage in bulk	fresh beefsteak
5		SHJ823	duck meat	Shanghai	Shanghai	20120905	Yunlian Fair	open-air for normal temperature	Live-bird
6		SHN837	chicken	Shanghai	Shanghai	20120905	Pusan Road Fair	open-air for normal temperature	Live-bird
7		SHN838	Bacon	Shanghai	Sichuan	N/A	Yunlian Fair	open-air for normal temperature	red Bacon
8		HFC851	pork	Hefei	N/A	N/A	Century Lianhua Supermarket	cold storage in bulk	fresh meat slices
9		HFC852	pork	Hefei	N/A	N/A	Century Lianhua Supermarket	cold storage in bulk	ground meat
10		HFC853	pork	Hefei	N/A	N/A	Ningguo Fair	open-air for normal temperature	ground meat
11		HFJ872	Beef	Hefei	N/A	N/A	Ningguo Fair	open-air for normal temperature	fresh meat slices
12		HFJ873	duck meat	Hefei	N/A	N/A	Xinghua Fair	open-air for normal temperature	Live-bird
13		HFJ887	chicken	Hefei	N/A	N/A	Zhong Fair	open-air for normal temperature	Live-bird
14		HFJ888	Bacon	Hefei	N/A	N/A	Century Lianhua Supermarket	cold storage in bulk	
15		NCC901	pork	Nanchang	Nanchang	20120923	Wal-Mart	cold storage in bulk	meat loaf
16		NCC902	pork	Nanchang	Nanchang	20120923	RT-Mart	cold storage in bulk	minced meat
17		NCC903	pork	Nanchang	Nanchang	20120923	Wal-Mart	cold storage in bulk	ground meat
18		NCJ922	mutton	Nanchang	Nanchang	20120923	Dunzitang Fair	Hanging in ormal temperature	meat loaf
19		NCJ923	duck meat	Nanchang	Nanchang	20120923	Dunzitang Fair	pen stack in normal temperatur	Live-bird
20		NCN937	chicken	Nanchang	Nanchang	N/A	Xi'mazhuang Fair	normal storage in bulk	Live-bird
21		NCN938	Bacon	Nanchang	Nanchang	N/A	Dunzitang Fair	normal storage in bulk	white bacon loaf
22		WHC951	pork	Wuhan	Hubei	20121009	Wal-Mart	cold storage in bulk	raw meat loaf
23		WHC952	pork	Wuhan	Hubei	20121009	Wal-Mart	cold storage in bulk	raw minced meat
24		WHC953	pork	Wuhan	Hubei	20121009	Lotus	cold storage in bulk	raw minced meat
25		WHJ972	mutton	Wuhan	Wuhan	N/A	Tujialing Raw Fair	open-air for normal temperature	fresh meat slices
26		WHJ973	duck meat	Wuhan	Wuhan	20121009	Tujialing Raw Fair	open-air for normal temperature	Live-bird
27		WHN987	chicken	Wuhan	Wuhan	20121009	Tujialing Raw Fair	open-air for normal temperature	Live-bird
28		WHN988	Bacon	Wuhan	Wuhan	N/A	Wutaizha market	cold storage in bulk	antonese style sausage
29		CDC1001	pork	Chengdu	Chengdu	20121016	Yankou people's shopping malls	cold storage in bulk	meat loaf
30		CDC1002	pork	Chengdu	Chengdu	20121016	Haolaiwu household mall	cold storage in bulk	raw minced meat
31		CDC1003	pork	Chengdu	Chengdu	20121016	Yankou people's shopping malls	cold storage in bulk	raw minced meat
32		CDJ1022	mutton	Chengdu	Chengdu	20121016	Bluestone bridge market	Hanging in ormal temperature	raw meat loaf
33		CDJ1023	duck meat	Chengdu	Chengdu	N/A	Haolaiwu household mall	cold storage in bulk	raw meat loaf
34		CDN1037	chicken	Chengdu	Chengdu	20121016	Xiaojiahe market	Hanging in ormal temperature	raw meat loaf
35		CDN1038	Bacon	Chengdu	Chengdu	N/A	farmer's markets	normal storage in bulk	white bacon loaf
36		KMC1051	pork	Kunming	Kunming	2012.11.04	Carrefour	cold storage in bulk	N/A
37		KMC1052	pork	Kunming	Kunming	2012.11.04	Carrefour	cold storage in bulk	N/A
38		KMC1053	pork	Kunming	Kunming	2012.11.04	Wal-Mart	cold storage in bulk	N/A

39	KMJ1072	mutton	Kunming	Kunming	2012.11.04	Tianyuanli yintan farmer's markets	Hanging in ormal temperature	N/A
40	KMJ1073	duck meat	Kunming	Kunming	2012.11.04	Tianyuanli yintan farmer's markets	cold storage in bulk	Live-bird
41	KMN1087	chicken	Kunming	Kunming	2012.11.04	Donghua comprehensive market	Hanging in ormal temperature	Live-bird
42	KMN1088	beef	Kunming	Kunming	2012.11.04	Tianyuanli yintan farmer's markets	normal storage in bulk	N/A
43	LZC1101	pork	Lanzhou	Lanzhou	20121111	China Resources Vanguard	cold storage in bulk	meat loaf
44	LZC1102	pork	Lanzhou	Lanzhou	20121111	Lanzhou Hualian Supermarket	cold storage in bulk	raw ground meat
45	LZC1103	pork	Lanzhou	Lanzhou	20121111	Lanzhou Hualian Supermarket	cold storage in bulk	raw ground meat
46	LZJ1122	mutton	Lanzhou	Lanzhou	20121111	Zhangye Road market	cold storage in bulk	loaf
47	LZJ1123	duck meat	Lanzhou	Lanzhou	N/A	Zhangye Road market	cold storage in bulk	Live-bird
48	LZN1137	chicken	Lanzhou	Lanzhou	20121111	Green market	open-air for normal temperature	Live-bird
49	LZN1138	Bacon	Lanzhou	Sichuan	N/A	Zhangsutan market	cold storage in bulk	yellow Bacon
50	HEBC1151	pork	Haerbin	Haerbin	2012.11.20	Wal-Mart	cold storage in bulk	N/A
51	HEBC1152	beef	Haerbin	Haerbin	2012.11.20	Wal-Mart	cold storage in bulk	N/A
52	HEBC1153	pork	Haerbin	Haerbin	2012.11.20	Wal-Mart	cold storage in bulk	N/A
53	HEBJ1172	beef	Haerbin	Haerbin	2012.11.20	Hada market	cold storage in bulk	N/A
54	HEBJ1173	chicken	Haerbin	Haerbin	2012.11.20	Hada market	cold storage in bulk	N/A
55	HEBN1187	chicken	Haerbin	Haerbin	2012.11.20	Daoli market	Hanging in ormal temperature	N/A
56	HEBN1188	pork	Haerbin	Haerbin	2012.11.20	Daoli market	cold storage in bulk	N/A
57	XAC1201	pork	Xi'an	Xi'an	2012.11.29	China Resources Vanguard	cold storage in bulk	N/A
58	XAC1202	pork	Xi'an	Xi'an	2012.11.29	Wal-Mart	cold storage in bulk	N/A
59	XAC1203	pork	Xi'an	Xi'an	2012.11.29	China Resources Vanguard	cold storage in bulk	N/A
60	XAJ1222	mutton	Xi'an	Xi'an	2012.11.29	three trees fruit and vegetable market	Hanging in ormal temperature	N/A
61	XAJ1223	duck meat	Xi'an	Xi'an	2012.11.29	Xianxin aquatic market	open-air for normal temperature	Live-bird
62	XAN1237	chicken	Xi'an	Xi'an	2012.11.29	Wangjiacun Fair	open-air for normal temperature	N/A
63	XAN1238	Bacon	Xi'an	Xi'an	2012.11.29	Xianxin aquatic market	open-air for normal temperature	N/A
64	TYC1251	pork	Taiyuan	Taiyuan	20121211	Meitehao supermarket	cold storage in bulk	N/A
65	TYC1252	pork	Taiyuan	Taiyuan	20121211	Wal-Mart	cold storage in bulk	N/A
66	TYC1253	pork	Taiyuan	Taiyuan	20121211	Meitehao supermarket	cold storage in bulk	N/A
67	TYJ1272	mutton	Taiyuan	Taiyuan	20121211	Upwell Supermarket	Hanging in ormal temperature	N/A
68	TYJ1273	duck meat	Taiyuan	Taiyuan	20121211	Dongan road comprehensive trade marke	cold storage in bulk	N/A
69	TYN1287	chicken	Taiyuan	Taiyuan	20121211	Dongan road comprehensive trade marke	Hanging in ormal temperature	N/A
70	TYN1288	Bacon	Taiyuan	Taiyuan	20121211	Upwell Supermarket	normal storage in bulk	
71	BJC1301	pork	Beijing	Beijing	12.12.18	Wal-Mart	cold storage in bulk	N/A
72	BJC1302	pork	Beijing	Beijing	12.12.18	Carrefour	cold storage in bulk	N/A
73	BJC1303	pork	Beijing	Beijing	12.12.18	Wal-Mart	cold storage in bulk	N/A
74	BJJ1322	mutton	Beijing	Beijing	N/A	Nongguangli Fair	cold storage in bulk	N/A
75	BJJ1323	duck meat	Beijing	Beijing	N/A	Nongguangli Fair	cold storage in bulk	N/A
76	BJN1337	chicken	Beijing	Beijing	12.12.18	Dingsheng market	open-air for normal temperature	N/A
77	BJN1338	Bacon	Beijing	Beijing	N/A	Dingsheng market	open-air for normal temperature	N/A
78	JNC1351	pork	Jinan	Jinan	N/A	TESCO	cold storage in bulk	big meat loaf

79		JNC1352	pork	Jinan	Jinan	20121225	Wal-Mart	cold storage in bulk	ground meat
80		JNC1353	pork	Jinan	Jinan	20121225	TESCO	cold storage in bulk	ground meat
81		JNJ1372	mutton	Jinan	N/A	N/A	Minghu market	cold storage in bulk	big meat loaf
82		JNJ1373	duck meat	Jinan	N/A	N/A	Quancheng Road market	cold storage in bulk	N/A
83		JNN1387	chicken	Jinan	N/A	N/A	Utama	cold storage in bulk	Live
84		JNN1388	sausage	Jinan	N/A	N/A	Minghu market	cold storage in bulk	Black
85		YXC1401	pork	Guangzhou	N/A	N/A	Wal-market	cold storage in bulk	fresh meat slices
86		YXC1402	beef	Guangzhou	N/A	N/A	Tesco	cold storage in bulk	minced meat
87		YXC1403	pork	Guangzhou	N/A	N/A	Wal-market	cold storage in bulk	minced meat
88		YXJ1422	pork	Guangzhou	N/A	N/A	Huifu new street Fair	normal storage in bulk	fresh meat slices
89		YXJ1423	chicken	Guangzhou	N/A	N/A	Shaheding market	normal storage in bulk	Live
90		YXJ1424	duck meat	Guangzhou	N/A	N/A	Huifu new street Fair	normal storage in bulk	Live
91		YXJ1425	Bacon	Guangzhou	Jiangmen	N/A	Shaheding market	normal storage in bulk	pocessed
92		LWC1451	pork	Guangzhou	Guangzhou	20130312	Parknshop	cold storage in bulk	N/A
93		LWC1452	pork	Guangzhou	Guangzhou	20130312	Carrefour	cold storage in bulk	N/A
94		LWC1453	pork	Guangzhou	Guangzhou	20130312	Parknshop	cold storage in bulk	N/A
95		LWN1472	pork	Guangzhou	Guangzhou	20130312	Zilai meat market	normal storage in bulk	N/A
96		LWN1473	duck meat	Guangzhou	Guangzhou	20130312	Parknshop	normal storage in bulk	N/A
97		LWN1474	duck meat	Guangzhou	Guangzhou	20130312	Zilai meat market	normal storage in bulk	N/A
98		LWN1475	pork	Guangzhou	Guangzhou	20130312	Longjin middle road Hongfu market	normal storage in bulk	N/A
99	**meat and meat product**	PYC1501	pork	Guangzhou	Guangzhou	20130319	China Resources Vanguard	cold storage in bulk	N/A
100		PYC1502	pork	Guangzhou	Guangzhou	20130319	Parknshop	cold storage in bulk	N/A
101		PYC1503	pork	Guangzhou	Guangzhou	20130319	China Resources Vanguard	cold storage in bulk	N/A
102		PYN1522	pork	Guangzhou	Guangzhou	20130319	Rainbow meat vegetable market	normal storage in bulk	N/A
103		PYN1523	duck meat	Guangzhou	Guangzhou	20130319	Jushu meat vegetable market	normal storage in bulk	N/A
104		PYN1524	chicken	Guangzhou	Guangzhou	20130319	Rainbow meat vegetable market	normal storage in bulk	N/A
105		PYN1525	pork	Guangzhou	Guangzhou	20130319	Jushu meat vegetable market	normal storage in bulk	N/A
106		CHC1551	pork	Guangzhou	N/A	N/A	Wanhe shopping mall	cold storage in bulk	fresh meat slices
107		CHC1552	beef	Guangzhou	N/A	N/A	Yonghenglong west street supermarket	cold storage in bulk	fresh meat slices
108		CHC1553	pork	Guangzhou	N/A	N/A	Wanhe shopping mall	cold storage in bulk	minced meat
109		CHJ1572	pork	Guangzhou	N/A	N/A	west street market	normal storage in bulk	fresh meat slices
110		CHJ1573	chicken	Guangzhou	N/A	N/A	Hedong meat vegetable market	normal storage in bulk	Live-bird
111		CHJ1574	duck meat	Guangzhou	N/A	N/A	west street market	normal storage in bulk	Live-bird
112		CHJ1575	sausage	Guangzhou	N/A	N/A	Hedong meat vegetable market	normal storage in bulk	pocessed
113		ZCC1601	pork	Guangzhou	Zengcheng	N/A	Renrenle Zengcheng shopping mall	open-air for normal temperature	packing
114		ZCC1602	beef	Guangzhou	Zengcheng	N/A	RT-Mart	cold storage in bulk	N/A
115		ZCC1603	pork	Guangzhou	Zengcheng	N/A	Renrenle Zengcheng shopping mall	open-air for normal temperature	N/A
116		ZCJ1622	pork	Guangzhou	Zengcheng	N/A	Licheng east Fair	open-air for normal temperature	N/A
117		ZCJ1623	chicken	Guangzhou	Zengcheng	N/A	Xiajie Fair	open-air for normal temperature	Live-bird
118		ZCJ1624	duck meat	Guangzhou	Zengcheng	N/A	Licheng east Fair	open-air for normal temperature	Live-bird

119	ZCJ1625	Bacon	Guangzhou	Zengcheng	N/A	Xiajie Fair	normal storage in bulk	packing
120	SZC1651	pork	Shenzhen	N/A	N/A	China Resources Vanguard	cold storage in bulk	N/A
121	SZC1652	beef	Shenzhen	N/A	N/A	Xinyijia	cold storage in bulk	N/A
122	SZC1653	pork	Shenzhen	N/A	N/A	China Resources Vanguard	cold storage in bulk	N/A
123	SZJ1672	pork	Shenzhen	N/A	N/A	Mingli market	normal storage in bulk	N/A
124	SZJ1673	chicken	Shenzhen	N/A	N/A	Hubei east market	normal storage in bulk	N/A
125	SZJ1674	duck meat	Shenzhen	N/A	N/A	Hubei east market	normal storage in bulk	N/A
126	SZJ1675	Bacon	Shenzhen	N/A	N/A	Hubei east market	packing	N/A
127	STC1701	pork	Shantou	N/A	N/A	Lotus	N/A	N/A
128	STC1702	beef	Shantou	N/A	N/A	Wal-Mart	N/A	N/A
129	STC1703	pork	Shantou	N/A	N/A	Wal-Mart	N/A	N/A
130	STJ1722	pork	Shantou	N/A	N/A	Donghu street market	N/A	N/A
131	STJ1723	chicken	Shantou	N/A	N/A	Donghu street market	N/A	N/A
132	STJ1724	duck meat	Shantou	N/A	N/A	Longkou north road market	N/A	N/A
133	STJ1725	sausage	Shantou	N/A	N/A	Longkou north road market	N/A	N/A
134	ZJC1751	pork	Zhanjiang	Zhanjiang	20130526	Wal-Mart	cold storage in bulk	N/A
135	ZJC1752	beef	Zhanjiang	Zhanjiang	20130526	Wal-Mart	cold storage in bulk	N/A
136	ZJC1753	pork	Zhanjiang	Zhanjiang	20130526	Aihua supermarket	cold storage in bulk	N/A
137	ZJJ1772	pork	Zhanjiang	Zhanjiang	20130526	Cikeng south market	normal storage in bulk	N/A
138	ZJJ1773	chicken	Zhanjiang	Zhanjiang	20130526	Cikeng north bridge market	normal storage in bulk	N/A
139	ZJJ1774	duck meat	Zhanjiang	Zhanjiang	20130526	Cikeng south market	normal storage in bulk	N/A
140	ZJJ1775	Bacon	Zhanjiang	Zhanjiang	20130506	Cikeng north bridge market	normal storage in bulk	N/A
141	SGC1801	pork	Shaoguan	Shaoguan	N/A	Wal-Mart	cold storage in bulk	meat loaf
142	SGC1802	beef	Shaoguan	Shaoguan	N/A	RT-Mart	cold storage in bulk	sliced meat
143	SGC1803	pork	Shaoguan	Shaoguan	N/A	Wal-Mart	cold storage in bulk	minced meat
144	SGJ1822	pork	Shaoguan	Shaoguan	N/A	Xinglong market	open-air for normal temperature	meat loaf
145	SGJ1823	chicken	Shaoguan	Shaoguan	N/A	Fengcai market	open-air for normal temperature	live
146	SGJ1824	duck meat	Shaoguan	Shaoguan	N/A	Xinglong market	open-air for normal temperature	live
147	SGJ1825	Bacon	Shaoguan	Shaoguan	N/A	Fengcai market	open-air for normal temperature	process
148	HYC1851	pork	Heyuan	Heyuan	N/A	Guangsheng market	cold storage in bulk	meat loaf
149	HYC1852	beef	Heyuan	Heyuan	N/A	Renrenle supermarket	cold storage in bulk	loaf
150	HYC1853	chicken	Heyuan	Heyuan	N/A	Guangsheng market	cold storage in bulk	ground meat
151	HYJ1872	pork	Heyuan	Heyuan	N/A	Yuancheng center market	open-air for normal temperature	loaf
152	HYJ1873	chicken	Heyuan	Heyuan	N/A	Xingyuan market	open-air for normal temperature	live
153	HYJ1874	duck meat	Heyuan	Heyuan	N/A	Yuancheng center market	open-air for normal temperature	live
154	HYJ1875	sausage	Heyuan	Heyuan	N/A	Xingyuan market	open-air for normal temperature	N/A
155	FZC2201	pork	Fuzhou	Fuzhou	N/A	Carrefour	packaging in low temperature	N/A
156	FZC2202	beef	Fuzhou	Fuzhou	N/A	Wal-Mart	packaging in low temperature	N/A
157	FZC2203	pork	Fuzhou	Fuzhou	N/A	Carrefour	packaging in low temperature	N/A
158	FZJ2222	pork	Fuzhou	Fuzhou	N/A	Xiyingli market	open-air in normal temperature	N/A

159		FZJ2223	chicken	Fuzhou	Fuzhou	N/A	Xiyingli market	open-air in normal temperature	N/A
160		FZJ2224	duck meat	Fuzhou	Fuzhou	N/A	Xiyingli market	open-air in normal temperature	N/A
161		FZJ2225	sausage	Fuzhou	Fuzhou	N/A	Xiyingli market	open-air in normal temperature	N/A
162		NNC2251	pork	Nanning	Nanning	N/A	China Resources Vanguard	cold storage in bulk	meat loaf
163		NNC2252	beef	Nanning	Nanning	N/A	Wal-Mart	cold storage in bulk	beef loaf
164		NNC2253	pork	Nanning	Nanning	N/A	China Resources Vanguard	cold storage in bulk	ground meat
165		NNJ2272	pork	Nanning	Nanning	N/A	Dancun market	open-air for normal temperature	meat loaf
166		NNJ2273	chicken	Nanning	Nanning	N/A	Dancun market	open-air for normal temperature	Live
167		NNJ2274	duck meat	Nanning	Nanning	N/A	Dancun market	open-air for normal temperature	Live
168		NNJ2275	sausage	Nanning	Nanning	N/A	Dancun market	open-air for normal temperature	process
169		XMC2301	pork	Xiamen	N/A	N/A	Wal-Mart	cold storage in bulk	meat loaf
170		XMC2302	beef	Xiamen	N/A	N/A	Tesco	cold storage in bulk	meat loaf
171		XMC2303	pork	Xiamen	N/A	N/A	Wal-Mart	cold storage in bulk	minced meat
172		XMJ2322	pork	Xiamen	N/A	N/A	The eight market	open-air for normal temperature	loaf
173		XMJ2323	chicken	Xiamen	N/A	N/A	Ruijing market	open-air for normal temperature	fresh
174		XMJ2324	duck meat	Xiamen	N/A	N/A	The eight market	open-air for normal temperature	fresh
175		XMJ2325	beef	Xiamen	N/A	N/A	Ruijing market	open-air for normal temperature	loaf
176		BHC2351	pork	Beihai	N/A	N/A	RT-Mart	cold storage in bulk	meat loaf
177		BHC2352	beef	Beihai	N/A	N/A	Hean supermarket	cold storage in bulk	loaf
178		BHC2353	pork	Beihai	N/A	N/A	RT-Mart	cold storage in bulk	minced meat
179		BHJ2372	pork	Beihai	N/A	N/A	Guizhou road market	open-air for normal temperature	loaf
180		BHJ2373	chicken	Beihai	N/A	N/A	Beijing road market	open-air for normal temperature	fresh
181		BHJ2374	duck meat	Beihai	N/A	N/A	Guizhou road market	open-air for normal temperature	fresh
182		BHJ2375	sausage	Beihai	N/A	N/A	Beijing road market	open-air for normal temperature	open-air
183		HKC2401	pork	Haikou	N/A	N/A	Parknshop	cold storage in bulk	meat loaf
184		HKC2402	beef	Haikou	N/A	N/A	Carrefour	cold storage in bulk	loaf
185		HKC2403	pork	Haikou	N/A	N/A	Parknshop	cold storage in bulk	meat minced
186		HKJ2422	pork	Haikou	N/A	N/A	Longshepo market	open-air for normal temperature	meat loaf
187		HKJ2423	chicken	Haikou	N/A	N/A	Mingsheng market	open-air for normal temperature	fresh
188		HKJ2424	duck meat	Haikou	N/A	N/A	Longshepo market	open-air for normal temperature	fresh
189		HKJ2425	sausage	Haikou	N/A	N/A	Mingsheng market	open-air for normal temperature	in bulk
190		SYC2451	pork	Sanya	N/A	N/A	Haowang supermarket	in low temperature	N/A
191		SYC2452	beef	Sanya	N/A	N/A	Fuleduo supermarket	in low temperature	N/A
192		SYC2453	pork	Sanya	N/A	N/A	Haowang supermarket	in low temperature	N/A
193		SYJ2472	pork	Sanya	N/A	N/A	The one market	normal temperature in bulk	N/A
194		SYJ2473	chicken	Sanya	N/A	N/A	City center market	normal temperature in bulk	N/A
195		SYJ2474	duck meat	Sanya	N/A	N/A	The one market	normal temperature in bulk	N/A
196		SYJ2475	sausage	Sanya	N/A	N/A	City center market	processed	N/A
197		SHC804	prawn	Shanghai	N/A	N/A	Tesco	pool in normal temperature	Live
198		SHC805	prawn	Shanghai	Shanghai	N/A	Carrefour	cold storage in bulk	Chilled
199		SHC806	yellow croaker	Shanghai	N/A	N/A	Tesco	cold storage in bulk	Chilled

200	SHC807	sleeve-fish	Shanghai	N/A	N/A	Carrefour	cold storage in bulk	Chilled
201	SHJ824	oyster meat	Shanghai	N/A	N/A	Hengda aquatic wholesale market	lowl temperature in sealed	husked in water
202	SHJ825	saury	Shanghai	N/A	N/A	Pusan Road market	normal storage in bulk	Chilled
203	SHJ826	grass carp	Shanghai	N/A	N/A	Pusan Road market	normal storage in bulk	Live
204	SHN839	grass carp	Shanghai	Shanghai	20120905	Pusan Road market	normal storage in bulk	Live
205	SHN840	Tilapia mossambica	Shanghai	Shanghai	20120905	Pusan Road market	pool in normal temperature	Live
206	SHN841	yellow croaker	Shanghai	N/A	N/A	Pusan Road market	pool in normal temperature	Live
207	HFC854	prawn	Hefei	N/A	N/A	Century Lianhua Supermarket	pool in normal temperature	Live
208	HFC855	shrimp	Hefei	N/A	N/A	Carrefour	pool in normal temperature	Live
209	HFC856	crucian	Hefei	N/A	N/A	Century Lianhua Supermarket	pool in normal temperature	Live
210	HFC857	sleeve-fish	Hefei	N/A	N/A	Carrefour	on ice	N/A
211	HFJ874	small cuttlefish	Hefei	N/A	N/A	Carrefour	on ice	N/A
212	HFJ875	weever	Hefei	N/A	N/A	Haozhou road market	on ice	N/A
213	HFJ876	grass carp	Hefei	N/A	N/A	Haozhou road market	in water	live
214	HFJ889	grass carp	Hefei	N/A	N/A	Zhong market	in water	live
215	HFJ890	crucian	Hefei	N/A	N/A	Ninglu road market	in water	live
216	HFJ891	Pelteobagrus fulvidraco	Hefei	N/A	N/A	Xinghua market	in water	live
217	NCC904	shrimp	Nanchang	N/A	N/A	Nanchang aquatic wholesale market	pool in normal temperature	Live
218	NCC905	prawn	Nanchang	N/A	N/A	Wal-Mart	pool in normal temperature	Live
219	NCC906	crucian	Nanchang	Nanchang	20120923	RT-Mart	pool in normal temperature	Live
220	NCC907	sleeve-fish	Nanchang	N/A	N/A	Wal-Mart	cold storage in bulk	Chilled
221	NCJ924	sleeve-fish	Nanchang	N/A	N/A	Dunzitang Fair	normal temperature in bulk	Chilled
222	NCJ925	saury	Nanchang	N/A	N/A	Dunzitang Fair	Frozen in bulk	Chilled
223	NCJ926	grass carp	Nanchang	Nanchang	N/A	Dunzitang Fair	pool in normal temperature	Live
224	NCN939	grass carp	Nanchang	Nanchang	N/A	Xi'mazhuang Fair	pool in normal temperature	Live
225	NCN940	Tilapia mossambica	Nanchang	Nanchang	N/A	Dunzitang Fair	pool in normal temperature	Live
226	NCN941	weever	Nanchang	N/A	N/A	Dunzitang Fair	pool in normal temperature	Live
227	WHC954	shrimp	Wuhan	N/A	N/A	Lotus	cold storage in bulk	Chilled
228	WHC955	shrimp	Wuhan	N/A	20121009	Wal-Mart	pool in normal temperature	Chilled
229	WHC956	crucian	Wuhan	N/A	20121009	Wal-Mart	pool in normal temperature	Live
230	WHC957	sleeve-fish	Wuhan	N/A	N/A	Lotus	cold storage in bulk	Chilled
231	WHJ974	sleeve-fish	Wuhan	Wuhan	N/A	Tujialing Raw Fair	pool in normal temperature	Wash
232	WHJ975	crucian	Wuhan	Wuhan	20121009	Tujialing Raw Fair	pool in normal temperature	Wash
233	WHJ976	grass carp	Wuhan	Wuhan	20121009	Tujialing Raw Fair	pool in normal temperature	Wash
234	WHN989	cabezon	Wuhan	Wuhan	20121009	Yushun meat market	pool in normal temperature	Wash
235	WHN990	mandarin fish	Wuhan	Wuhan	20121009	Yushun meat market	pool in normal temperature	Wash
236	WHN991	weever	Wuhan	Wuhan	20121009	Yushun meat market	pool in normal temperature	Wash
237	CDC1004	shrimp	Chengdu	Chengdu	20121016	Bluestone bridge market	pool in normal temperature	Live
238	CDC1005	prawn	Chengdu	Chengdu	20121016	Bluestone bridge market	pool in normal temperature	Live
239	CDC1006	crucian	Chengdu	Chengdu	20121016	Yankou people's shopping malls	pool in normal temperature	Live
240	CDC1007	sleeve-fish	Chengdu	N/A	N/A	Bluestone bridge market	cold storage in bulk	Chilled

241	CDJ1024	sleeve-fish	Chengdu	N/A	N/A	Bluestone bridge market	normal storage in bulk	Chilled
242	CDJ1025	checkmate fish	Chengdu	Chengdu	20121016	Bluestone bridge market	pool in normal temperature	Live
243	CDJ1026	grass carp	Chengdu	Chengdu	20121016	farmer's markets	pool in normal temperature	Live
244	CDN1039	grass carp	Chengdu	Chengdu	20121016	Xiaojiahe market	pool in normal temperature	Live
245	CDN1040	crucian	Chengdu	Chengdu	20121016	Randeng market	pool in normal temperature	Live
246	CDN1041	weever	Chengdu	Chengdu	20121016	Randeng market	pool in normal temperature	Live
247	KMC1054	shrimp	Kunming	Kunming	2012.11.04	Carrefour	cold storage in bulk	N/A
248	KMC1055	prawn	Kunming	Kunming	2012.11.04	Wal-Mart	cold storage in bulk	N/A
249	KMC1056	yellow croaker	Kunming	Kunming	2012.11.04	Wal-Mart	cold storage in bulk	N/A
250	KMC1057	sleeve-fish	Kunming	Kunming	2012.11.04	Carrefour	cold storage in bulk	N/A
251	KMJ1074	sleeve-fish	Kunming	Kunming	2012.11.04	Wal-Mart	cold storage in bulk	N/A
252	KMJ1075	saury	Kunming	Kunming	2012.11.04	Tianyuanli yintan farmer's markets	pool in normal temperature	N/A
253	KMJ1076	grass carp	Kunming	Kunming	2012.11.04	Donghua comprehensive market	pool in normal temperature	N/A
254	KMN1089	grass carp	Kunming	Kunming	2012.11.04	Tianyuanli yintan farmer's markets	pool in normal temperature	N/A
255	KMN1090	Tilapia mossambica	Kunming	Kunming	2012.11.04	Tianyuanli yintan farmer's markets	pool in normal temperature	N/A
256	KMN1091	yellow croaker	Kunming	Kunming	2012.11.04	Tianyuanli yintan farmer's markets	pool in normal temperature	N/A
257	LZC1104	shrimp	Lanzhou	N/A	N/A	Lanzhou Hualian Supermarket	cold storage in bulk	Fresh
258	LZC1105	prawn	Lanzhou	N/A	N/A	China Resources Vanguard	cold storage in bulk	Fresh
259	LZC1106	crucian	Lanzhou	Lanzhou	N/A	Lanzhou Hualian Supermarket	pool in normal temperature	Live
260	LZC1107	sleeve-fish	Lanzhou	N/A	N/A	Lanzhou Hualian Supermarket	cold storage in bulk	Chilled
261	LZJ1124	sleeve-fish	Lanzhou	N/A	N/A	Bulan road street market	cold storage in bulk	Chilled
262	LZJ1125	saury	Lanzhou	N/A	N/A	Zhangye Road market	cold storage in bulk	Chilled
263	LZJ1126	grass carp	Lanzhou	N/A	20121111	Zhangye Road market	pool in cold temperature	Live
264	LZN1139	grass carp	Lanzhou	N/A	20121111	Bulan road street market	pool in cold temperature	Live
265	LZN1140	cyprinoid	Lanzhou	N/A	20121111	Bulan road street market	pool in cold temperature	Live
266	LZN1141	oyster meat	Lanzhou	N/A	N/A	Bulan road street market	pool in cold temperature	shelled
267	HEBC1154	shrimp	Haerbin	Haerbin	2012.11.20	Wal-Mart	pool in normal temperature	N/A
268	HEBC1155	prawn	Haerbin	N/A	2012.11.20	Zhongyanghong supermarket	pool in normal temperature	N/A
269	HEBC1156	crucian	Haerbin	Haerbin	2012.11.20	Wal-Mart	pool in normal temperature	N/A
270	HEBC1157	sleeve-fish	Haerbin	N/A	2012.11.20	Wal-Mart	cold storage in bulk	N/A
271	HEBJ1174	sleeve-fish	Haerbin	N/A	N/A	Hada aquatic wholesale market	cold storage in bulk	N/A
272	HEBJ1175	yellow croaker	Haerbin	N/A	2012.11.20	Hada aquatic wholesale market	pool in normal temperature	N/A
273	HEBJ1176	crucian	Haerbin	Haerbin	2012.11.20	Hada aquatic wholesale market	pool in normal temperature	N/A
274	HEBN1189	yellow croaker	Haerbin	N/A	2012.11.20	Daoli market	pool in normal temperature	N/A
275	HEBN1190	crucian	Haerbin	Haerbin	2012.11.20	Daoli market	pool in normal temperature	N/A
276	HEBN1191	oyster meat	Haerbin	Dalian	N/A	Daoli market	pool in normal temperature	N/A
277	XAC1204	prawn	Xi'an	Xi'an	2012.11.29	Wal-Mart	pool in normal temperature	N/A
278	XAC1205	prawn	Xi'an	Xi'an	2012.11.29	China Resources Vanguard	pool in normal temperature	N/A
279	XAC1206	Fish	Xi'an	Xi'an	2012.11.29	China Resources Vanguard	pool in normal temperature	N/A
280	XAC1207	cuttlefish	Xi'an	Xi'an	2012.11.29	Wal-Mart	on ice	N/A
281	XAJ1224	sleeve-fish	Xi'an	Xi'an	2012.11.29	Xianxin aquatic market	on ice	N/A

282	XAJ1225	dace	Xi'an	Xi'an	2012.11.29	Xianxin aquatic market	on ice	N/A
283	XAJ1226	grass carp	Xi'an	Xi'an	2012.11.29	Wangjiacun Fair	pool in normal temperature	N/A
284	XAN1239	grass carp	Xi'an	Xi'an	2012.11.29	Wangjiacun Fair	pool in normal temperature	N/A
285	XAN1240	blunt-snout bream	Xi'an	Xi'an	2012.11.29	Sankeshu Fair	pool in normal temperature	N/A
286	XAN1241	crucian	Xi'an	Xi'an	2012.11.29	Wangjiacun Fair	pool in normal temperature	N/A
287	TYC1254	prawn	Taiyuan	Taiyuan	20121211	Meitehao supermarket	pool in normal temperature	N/A
288	TYC1255	prawn	Taiyuan	Taiyuan	20121211	Wal-Mart	pool in normal temperature	N/A
289	TYC1256	yellow croaker	Taiyuan	Taiyuan	20121211	Wal-Mart	pool in normal temperature	N/A
290	TYC1257	sleeve-fish	Taiyuan	Taiyuan	N/A	Meitehao supermarket	cold storage in bulk	N/A
291	TYJ1274	sleeve-fish	Taiyuan	N/A	N/A	Taiyuan fruit north market	normal temperature in bulk	N/A
292	TYJ1275	crucian	Taiyuan	N/A	20121211	Taiyuan fruit north market	pool in normal temperature	N/A
293	TYJ1276	grass carp	Taiyuan	N/A	20121211	Taiyuan fruit north market	pool in normal temperature	N/A
294	TYN1289	grass carp	Taiyuan	Taiyuan	20121211	Upwell Supermarket	pool in normal temperature	N/A
295	TYN1290	Tilapia mossambica	Taiyuan	Taiyuan	20121211	Wulongkou seafood market	pool in normal temperature	N/A
296	TYN1291	cyprinoid	Taiyuan	Taiyuan	20121211	Wulongkou seafood market	pool in normal temperature	N/A
297	BJC1304	prawn	Beijing	N/A	N/A	Carrefour	pool in normal temperature	N/A
298	BJC1305	prawn	Beijing	N/A	N/A	Wal-Mart	pool in normal temperature	N/A
299	BJC1306	weever	Beijing	N/A	N/A	Wal-Mart	pool in normal temperature	N/A
300	BJC1307	sleeve-fish	Beijing	N/A	N/A	Carrefour	cold storage in bulk	N/A
301	BJJ1324	sleeve-fish	Beijing	N/A	N/A	Nongguangli Fair	cold storage in bulk	N/A
302	BJJ1325	crucian	Beijing	N/A	N/A	Nongguangli Fair	pool in normal temperature	N/A
303	BJJ1326	grass carp	Beijing	N/A	N/A	Nongguangli Fair	pool in normal temperature	N/A
304	BJN1339	grass carp	Beijing	N/A	N/A	Dingsheng market	pool in normal temperature	N/A
305	BJN1340	Tilapia mossambica	Beijing	N/A	N/A	Dingsheng market	pool in normal temperature	N/A
306	BJN1341	yellow croaker	Beijing	N/A	N/A	Dingsheng market	pool in normal temperature	N/A
307	JNC1354	prawn	Jinan	N/A	N/A	Wal-Mart	cold storage in bulk	Chilled
308	JNC1355	prawn	Jinan	N/A	N/A	TESCO	cold storage in bulk	Chilled
309	JNC1356	yellow croaker	Jinan	N/A	N/A	Wal-Mart	cold storage in bulk	Chilled
310	JNC1357	sleeve-fish	Jinan	N/A	N/A	TESCO	cold storage in bulk	Chilled
311	JNJ1374	sleeve-fish	Jinan	N/A	N/A	Zhangzhuang road Fair	cold storage in bulk	Chilled
312	JNJ1375	mackerel	Jinan	N/A	N/A	Minghu market	cold storage in bulk	Chilled
313	JNJ1376	grass carp	Jinan	Jinan	20121225	Minghu market	pool in cold temperature	Live
314	JNN1389	grass carp	Jinan	Jinan	20121225	Weiyi Road Fair	pool in cold temperature	Live
315	JNN1390	Tilapia mossambica	Jinan	N/A	N/A	N/A	N/A	N/A
316	JNN1391	yellow croaker	Jinan	N/A	N/A	Zhangzhuang road Fair	cold storage in bulk	Chilled
317	YXC1404	shrimp	Guangzhou	N/A	N/A	Tesco	pool in normal temperature	Live
318	YXC1405	prawn	Guangzhou	N/A	N/A	Wal-market	pool in normal temperature	Live
319	YXC1406	yellow croaker	Guangzhou	N/A	N/A	Tesco	cold storage in bulk	Chilled
320	YXC1407	sleeve-fish	Guangzhou	N/A	N/A	Wal-market	cold storage in bulk	Chilled
321	YXJ1426	oyster meat	Guangzhou	N/A	N/A	Huifu new street Fair	normal storage in bulk	shelled
322	YXJ1427	saury	Guangzhou	N/A	N/A	Shaheding market	normal storage in bulk	Chilled

323		YXJ1428	Pelteobagrus fulvidraco	Guangzhou	N/A	N/A	Huifu new street Fair	normal storage in bulk	Live
324		YXJ1429	grass carp	Guangzhou	N/A	N/A	Shaheding market	normal storage in bulk	Live
325		YXJ1430	crucian	Guangzhou	N/A	N/A	Huifu new street Fair	normal storage in bulk	Live
326		YXJ1431	Tilapia mossambica	Guangzhou	N/A	N/A	Shaheding market	normal storage in bulk	Live
327		LWC1454	snakehead	Guangzhou	N/A	20130312	Carrefour	pool in normal temperature	Live
328		LWC1455	crucian	Guangzhou	N/A	20130312	Carrefour	pool in normal temperature	Live
329		LWC1456	Tilapia mossambica	Guangzhou	Guangzhou	20130312	Parknshop	pool in normal temperature	Live
330		LWC1457	sleeve-fish	Guangzhou	Guangzhou	20130312	Parknshop	on ice	Chilled
331		LWN1476	Pelteobagrus fulvidraco	Guangzhou	Guangzhou	20130312	Zilai meat market	pool in normal temperature	Live
332		LWN1477	pond fish	Guangzhou	Guangzhou	20130312	Longjin middle road Hongfu market	pool in normal temperature	Live
333		LWN1478	crucian	Guangzhou	Guangzhou	20130312	Zilai meat market	pool in normal temperature	Live
334		LWN1479	grass carp	Guangzhou	Guangzhou	20130312	Longjin middle road Hongfu market	pool in normal temperature	Live
335		LWN1480	cabezon	Guangzhou	Guangzhou	20130312	Zilai meat market	pool in normal temperature	Live
336	**Aquatic products**	LWN1481	grass carp	Guangzhou	Guangzhou	20130312	Longjin middle road Hongfu market	pool in normal temperature	Live
337		PYC1504	pond fish	Guangzhou	Guangzhou	20130319	Parknshop	pool in normal temperature	Live
338		PYC1505	shrimp	Guangzhou	Guangzhou	20130319	China Resources Vanguard	pool in normal temperature	Live
339		PYC1506	Tilapia mossambica	Guangzhou	Guangzhou	20130319	Parknshop	pool in normal temperature	Live
340		PYC1507	Tilapia mossambica	Guangzhou	Guangzhou	20130319	China Resources Vanguard	pool in normal temperature	Live
341		PYN1526	sleeve-fish	Guangzhou	Guangzhou	20130319	Rainbow meat vegetable market	on ice	Chilled
342		PYN1527	sleeve-fish	Guangzhou	Guangzhou	20130319	Jushu meat vegetable market	on ice	Chilled
343		PYN1528	crucian	Guangzhou	Guangzhou	20130319	Rainbow meat vegetable market	pool in normal temperature	Live
344		PYN1529	crucian	Guangzhou	Guangzhou	20130319	Jushu meat vegetable market	pool in normal temperature	Live
345		PYN1530	cabezon	Guangzhou	Guangzhou	20130319	Rainbow meat vegetable market	pool in normal temperature	Live
346		PYN1531	shrimp	Guangzhou	Guangzhou	20130319	Jushu meat vegetable market	pool in normal temperature	Live
347		CHC1554	weever	Guangzhou	N/A	N/A	Yonghenglong west street supermarket	pool in normal temperature	Live
348		CHC1555	crucian	Guangzhou	N/A	N/A	Yonghenglong west street supermarket	pool in normal temperature	Live
349		CHC1556	Tilapia mossambica	Guangzhou	N/A	N/A	Yonghenglong west street supermarket	pool in normal temperature	Live
350		CHC1557	fish belly	Guangzhou	N/A	N/A	Yonghenglong west street supermarket	pool in normal temperature	Live
351		CHJ1576	sleeve-fish	Guangzhou	N/A	N/A	west street market	normal temperature	Live
352		CHJ1577	weever	Guangzhou	N/A	N/A	Hedong meat vegetable market	normal temperature	Live
353		CHJ1578	yellow croaker	Guangzhou	N/A	N/A	west street market	normal temperature	Live
354		CHJ1579	grass carp	Guangzhou	N/A	N/A	Hedong meat vegetable market	normal temperature	Live
355		CHJ1580	cabezon	Guangzhou	N/A	N/A	west street market	normal temperature	Live
356		CHJ1581	Tilapia mossambica	Guangzhou	N/A	N/A	Hedong meat vegetable market	normal temperature	Live
357		ZCC1604	shrimp	Guangzhou	Zhanjing	N/A	RT-Mart	pool in normal temperature	Live
358		ZCC1605	shrimp shrimp	Guangzhou	N/A	N/A	Renrenle Zengcheng shopping mall	pool in normal temperature	Live
359		ZCC1606	yellow croaker	Guangzhou	N/A	N/A	RT-Mart	pool in normal temperature	Live
360		ZCC1607	sleeve-fish	Guangzhou	N/A	N/A	Renrenle Zengcheng shopping mall	on ice	N/A
361		ZCJ1626	sleeve-fish	Guangzhou	N/A	N/A	Licheng east Fair	on ice	N/A
362		ZCJ1627	Capelin	Guangzhou	N/A	N/A	Xiajie Fair	on ice	N/A
363		ZCJ1628	saury	Guangzhou	N/A	N/A	Licheng east Fair	on ice	N/A

364	ZCJ1629	Fresh fish	Guangzhou	N/A	N/A	Xiajie Fair	pool in normal temperature	Live
365	ZCJ1630	crucian	Guangzhou	N/A	N/A	Licheng east Fair	pool in normal temperature	Live
366	ZCJ1631	Tilapia mossambica	Guangzhou	N/A	N/A	Xiajie Fair	pool in normal temperature	Live
367	SZC1654	shrimp	Shenzhen	N/A	N/A	Xinyijia	pool in normal temperature	Live
368	SZC1655	shrimp	Shenzhen	N/A	N/A	China Resources Vanguard	pool in normal temperature	Live
369	SZC1656	golden thread	Shenzhen	N/A	N/A	Xinyijia	on ice	N/A
370	SZC1657	sleeve-fish	Shenzhen	N/A	N/A	China Resources Vanguard	on ice	N/A
371	SZJ1676	sleeve-fish	Shenzhen	N/A	N/A	Hubei east market	on ice	N/A
372	SZJ1677	golden thread	Shenzhen	N/A	N/A	Hubei east market	pool in normal temperature	Live
373	SZJ1678	golden thread	Shenzhen	N/A	N/A	Mingli market	pool in normal temperature	Live
374	SZJ1679	crucian	Shenzhen	N/A	N/A	Hubei east market	pool in normal temperature	Live
375	SZJ1680	crucian	Shenzhen	N/A	N/A	Mingli market	pool in normal temperature	Live
376	SZJ1681	shrimp	Shenzhen	N/A	N/A	Hubei east market	pool in normal temperature	Live
377	STC1704	Tilapia mossambica	Shantou	N/A	N/A	Lotus	pool in normal temperature	Live
378	STC1705	sea fish	Shantou	N/A	N/A	Wal-Mart	with ice in bulk	N/A
379	STC1706	saury	Shantou	N/A	N/A	Lotus	with ice in bulk	N/A
380	STC1707	sleeve-fish	Shantou	N/A	N/A	Wal-Mart	with ice in bulk	N/A
381	STJ1726	oyster meat	Shantou	N/A	N/A	Donghu street market	with ice in bulk	N/A
382	STJ1727	cuttlefish	Shantou	N/A	N/A	Longkou north road market	pool in normal temperature	N/A
383	STJ1728	weever	Shantou	N/A	N/A	Longkou north road market	pool in normal temperature	N/A
384	STJ1729	grass carp	Shantou	N/A	N/A	Donghu street market	pool in normal temperature	N/A
385	STJ1730	crucian	Shantou	N/A	N/A	Donghu street market	pool in normal temperature	N/A
386	STJ1731	Aconitum fish	Shantou	N/A	N/A	Longkou north road market	pool in normal temperature	N/A
387	ZJC1754	sea shrimp	Zhanjiang	Zhanjiang	20130526	Nanhua market	normal temperature	N/A
388	ZJC1755	sleeve-fish	Zhanjiang	Zhanjiang	20130526	Wal-Mart	low temperature	N/A
389	ZJC1756	raw fish	Zhanjiang	Zhanjiang	20130526	Aihua supermarket	normal temperature	N/A
390	ZJC1757	Tilapia mossambica	Zhanjiang	Zhanjiang	20130526	Wal-Mart	normal temperature	N/A
391	ZJJ1776	sleeve-fish	Zhanjiang	Zhanjiang	20130526	Cikan Nanhua market	normal temperature	N/A
392	ZJJ1777	sleeve-fish	Zhanjiang	Zhanjiang	20130526	Cikan north bridge market	normal temperature	N/A
393	ZJJ1778	shrimp	Zhanjiang	Zhanjiang	20130526	Cikan Nanhua market	normal temperature	N/A
394	ZJJ1779	crucian	Zhanjiang	Zhanjiang	20130526	Cikan north bridge market	normal temperature	N/A
395	ZJJ1780	grass carp	Zhanjiang	Zhanjiang	20130526	Cikan Nanhua market	normal temperature	N/A
396	ZJJ1781	shrimp	Zhanjiang	Zhanjiang	20130526	Cikan north bridge market	normal temperature	N/A
397	SGC1804	shrimp	Shaoguan	N/A	N/A	RT-Mart	pool in normal temperature	N/A
398	SGC1805	crucian	Shaoguan	N/A	N/A	Wal-Mart	pool in normal temperature	N/A
399	SGC1806	Tilapia mossambica	Shaoguan	N/A	N/A	RT-Mart	pool in normal temperature	N/A
400	SGC1807	sleeve-fish	Shaoguan	N/A	N/A	Wal-Mart	low temperature	N/A
401	SGJ1826	sleeve-fish	Shaoguan	N/A	N/A	Xinglong market	low temperature	N/A
402	SGJ1827	prawn	Shaoguan	N/A	N/A	Fengcai market	pool in normal temperature	N/A
403	SGJ1828	crucian	Shaoguan	N/A	N/A	Xinglong market	pool in normal temperature	N/A
404	SGJ1829	fish	Shaoguan	N/A	N/A	Fengcai market	pool in normal temperature	N/A

405	SGJ1830	grass carp	Shaoguan	N/A	N/A	Xinglong market	pool in normal temperature	N/A
406	SGJ1831	Tilapia mossambica	Shaoguan	N/A	N/A	Fengcai market	pool in normal temperature	N/A
407	HYC1854	shrimp	Heyuan	N/A	N/A	Renrenle supermarket	pool in normal temperature	N/A
408	HYC1855	crucian	Heyuan	N/A	N/A	Guangsheng supermarket	pool in normal temperature	N/A
409	HYC1856	Tilapia mossambica	Heyuan	N/A	N/A	Renrenle supermarket	pool in normal temperature	N/A
410	HYC1857	sleeve-fish	Heyuan	N/A	N/A	Guangsheng supermarket	low temperature	N/A
411	HYJ1876	sleeve-fish	Heyuan	N/A	N/A	Yuancheng center market	low temperature	N/A
412	HYJ1877	prawn	Heyuan	N/A	N/A	Xingyuan market	pool in normal temperature	N/A
413	HYJ1878	crucian	Heyuan	N/A	N/A	Yuancheng center market	pool in normal temperature	N/A
414	HYJ1879	yellow croaker	Heyuan	N/A	N/A	Xingyuan market	pool in normal temperature	N/A
415	HYJ1880	Tilapia mossambica	Heyuan	N/A	N/A	Yuancheng center market	pool in normal temperature	N/A
416	HYJ1881	crucian	Heyuan	N/A	N/A	Xingyuan market	pool in normal temperature	N/A
417	FZC2204	shrimp	Fuzhou	N/A	N/A	Wal-Mart	pool in normal temperature	N/A
418	FZC2205	crucian	Fuzhou	N/A	N/A	Carrefour	pool in normal temperature	N/A
419	FZC2206	yellow croaker	Fuzhou	N/A	N/A	Wal-Mart	pool in normal temperature	N/A
420	FZC2207	sleeve-fish	Fuzhou	N/A	N/A	Carrefour	on ice	N/A
421	FZJ2226	sleeve-fish	Fuzhou	N/A	N/A	Xiyingli market	on ice	N/A
422	FZJ2227	prawn	Fuzhou	N/A	N/A	Xiyingli market	pool in normal temperature	N/A
423	FZJ2228	yellow croaker	Fuzhou	N/A	N/A	Xiyingli market	pool in normal temperature	N/A
424	FZJ2229	crucian	Fuzhou	N/A	N/A	Xiyingli market	pool in normal temperature	N/A
425	FZJ2230	grass carp	Fuzhou	N/A	N/A	Xiyingli market	pool in normal temperature	N/A
426	FZJ2231	Water Fish	Fuzhou	N/A	N/A	Xiyingli market	pool in normal temperature	N/A
427	NNC2254	shrimp	Nanning	N/A	N/A	Wal-Mart	pool in cold temperature	live
428	NNC2255	Tilapia mossambica	Nanning	N/A	N/A	China Resources Vanguard	pool in cold temperature	live
429	NNC2256	cuttlefish	Nanning	N/A	N/A	Wal-Mart	pool in cold temperature	ice
430	NNC2257	sleeve-fish	Nanning	N/A	N/A	China Resources Vanguard	pool in cold temperature	ice
431	NNJ2276	sleeve-fish	Nanning	N/A	N/A	Dancun market	open-air with ice	ice
432	NNJ2277	sleeve-fish	Nanning	N/A	N/A	Dancun market	open-air with ice	ice
433	NNJ2278	yellow croaker	Nanning	N/A	N/A	Dancun market	open-air with ice	live
434	NNJ2279	yellow croaker	Nanning	N/A	N/A	Dancun market	open-air with ice	live
435	NNJ2280	grass carp	Nanning	N/A	N/A	Dancun market	open-air with ice	live
436	NNJ2281	sea shrimp	Nanning	N/A	N/A	Dancun market	open-air with ice	live
437	XMC2304	cyprinoid	Xiamen	N/A	N/A	Tesco	poor in low temperature	N/A
438	XMC2305	crucian	Xiamen	N/A	N/A	Wal-Mart	poor in low temperature	N/A
439	XMC2306	cuttlefish	Xiamen	N/A	N/A	Tesco	on ice	N/A
440	XMC2307	sleeve-fish	Xiamen	N/A	N/A	Wal-Mart	on ice	N/A
441	XMJ2326	sleeve-fish	Xiamen	N/A	N/A	The eight market	on ice	N/A
442	XMJ2327	sleeve-fish	Xiamen	N/A	N/A	Ruijing market	on ice	N/A
443	XMJ2328	yellow croaker	Xiamen	N/A	N/A	The eight market	poor in low temperature	N/A
444	XMJ2329	yellow croaker	Xiamen	N/A	N/A	Ruijing market	poor in low temperature	N/A
445	XMJ2330	Tilapia mossambica	Xiamen	N/A	N/A	The eight market	poor in low temperature	N/A

446		XMJ2331	sea shrimp	Xiamen	N/A	N/A	Ruijing market	poor in low temperature	N/A
447		BHC2354	crucian	Beihai	N/A	N/A	Hean supermarket	pool in normal temperature	live
448		BHC2355	grass carp	Beihai	N/A	N/A	RT-Mart	pool in normal temperature	live
449		BHC2356	grass carp	Beihai	N/A	N/A	Hean supermarket	pool in normal temperature	live
450		BHC2357	sleeve-fish	Beihai	N/A	N/A	RT-Mart	cold storage in bulk	Chilled
451		BHJ2376	sea fish	Beihai	N/A	N/A	Guizhou road market	cold storage in bulk	Chilled
452		BHJ2377	white shrimp	Beihai	N/A	N/A	Beijing road market	cold storage in bulk	live
453		BHJ2378	leaf sole	Beihai	N/A	N/A	Guizhou road market	cold storage in bulk	Chilled
454		BHJ2379	cabezon	Beihai	N/A	N/A	Beijing road market	cold storage in bulk	live
455		BHJ2380	Tilapia mossambica	Beihai	N/A	N/A	Guizhou road market	cold storage in bulk	live
456		BHJ2381	Tilapia mossambica	Beihai	N/A	N/A	Beijing road market	pool in normal temperature	live
457		HKC2404	shrimp	Haikou	N/A	N/A	Carrefour	cold storage in bulk	dead
458		HKC2405	weever	Haikou	N/A	N/A	Parknshop	pool in normal temperature	live
459		HKC2406	Tilapia mossambica	Haikou	N/A	N/A	Carrefour	pool in normal temperature	live
460		HKC2407	sleeve-fish	Haikou	N/A	N/A	Parknshop	cold storage in bulk	Chilled
461		HKJ2426	west fish	Haikou	N/A	N/A	Longshepo market	cold storage in bulk	Chilled
462		HKJ2427	wild shrimp	Haikou	N/A	N/A	Mingsheng market	cold storage in bulk	fresh
463		HKJ2428	sleeve-fish	Haikou	N/A	N/A	Longshepo market	cold storage in bulk	Chilled
464		HKJ2429	Tilapia mossambica	Haikou	N/A	N/A	Mingsheng market	pool in normal temperature	live
465		HKJ2430	Tilapia mossambica	Haikou	N/A	N/A	Longshepo market	pool in normal temperature	live
466		HKJ2431	sleeve-fish	Haikou	N/A	N/A	Mingsheng market	cold storage in bulk	Chilled
467		SYC2454	whitebait	Sanya	N/A	N/A	Fuleduo supermarket	pool in normal temperature	N/A
468		SYC2455	white pomfret	Sanya	N/A	N/A	Fuleduo supermarket	pool in normal temperature	N/A
469		SYC2456	hairtail	Sanya	N/A	N/A	Fuleduo supermarket	pool in normal temperature	N/A
470		SYC2457	Nemipterus virgatus	Sanya	N/A	N/A	Fuleduo supermarket	pool in normal temperature	N/A
471		SYJ2476	sleeve-fish	Sanya	N/A	N/A	The one market	pool in normal temperature	N/A
472		SYJ2477	sea shrimp	Sanya	N/A	N/A	City center market	pool in normal temperature	N/A
473		SYJ2478	yellow croaker	Sanya	N/A	N/A	The one market	pool in normal temperature	N/A
474		SYJ2479	sleeve-fish	Sanya	N/A	N/A	City center market	pool in normal temperature	N/A
475		SYJ2480	cabezon	Sanya	N/A	N/A	The one market	pool in normal temperature	N/A
476		SYJ2481	red sword-tail	Sanya	N/A	N/A	City center market	pool in normal temperature	N/A
477		SHC816	dumpling	Shanghai	Zhejiang	20120812	Carrefour	Frozen with sealed	Frozen
478		SHC817	Meat dumplings	Shanghai	Shanghai	20120801	Tesco	Frozen with sealed	Frozen
479		SHC818	lentinus edodes meatballs	Shanghai	Shanghai	20120820	Tesco	Frozen with sealed	Frozen
480		SHJ832	frozen broiler wing	Shanghai	N/A	N/A	Pusan Road market	Frozen in bulk	Frozen
481		SHJ833	Frozen mutton	Shanghai	N/A	N/A	Shanghai Yunlian market	Frozen in bulk	Frozen
482		SHN847	frozen broiler wing	Shanghai	N/A	N/A	Liuli Fair	Frozen in bulk	Frozen
483		SHN848	frozen broiler leg	Shanghai	N/A	N/A	Liuli Fair	Frozen in bulk	Frozen
484		HFC866	dumpling	Hefei	N/A	N/A	Century Lianhua Supermarket	packing in freezer	Frozen
485		HFC867	dumpling	Hefei	N/A	N/A	Carrefour	packing in freezer	Frozen
486		HFC868	steamed bun stuffed	Hefei	N/A	N/A	Century Lianhua Supermarket	packing in freezer	Frozen

487	HFJ882	frozen broiler wing	Hefei	N/A	N/A	Xinghua market	Frozen in bulk	Frozen
488	HFJ883	Frozen mutton	Hefei	N/A	N/A	Zhong market	Frozen in bulk	Frozen
489	HFJ897	frozen broiler wing	Hefei	N/A	N/A	Zhong market	Frozen in bulk	Frozen
490	HFJ898	frozen broiler leg	Hefei	N/A	N/A	Haozhou road market	Frozen in bulk	Frozen
491	NCC916	dumpling	Nanchang	Guangdong	20120826	Wal-Mart	Frozen with sealed	Frozen
492	NCC917	dumpling	Nanchang	N/A	20120905	Wal-Mart	Frozen with sealed	Frozen
493	NCC918	steamed stuffed bun	Nanchang	Jiangxi	20120813	Wal-Mart	Frozen with sealed	Frozen
494	NCJ932	frozen broiler leg	Nanchang	N/A	N/A	Dunzitang Fair	cold storage in bulk	Frozen
495	NCJ933	frozen beef	Nanchang	N/A	N/A	Dunzitang Fair	Frozen in bulk	Frozen
496	NCN947	frozen broiler wing	Nanchang	N/A	N/A	Xi'mazhuang Fair	Frozen in bulk	Frozen
497	NCN948	frozen broiler leg	Nanchang	N/A	N/A	Xi'mazhuang Fair	Frozen in bulk	Frozen
498	WHC966	smashed bean bun	Wuhan	Hubei	20121001	Lotus	Frozen with sealed	Frozen
499	WHC967	litter dumpling	Wuhan	Hubei	20121005	Wal-Mart	Frozen with sealed	Frozen
500	WHC968	dumpling	Wuhan	Hubei	20121006	Lotus	Frozen with sealed	Frozen
501	WHJ982	frozen broiler leg	Wuhan	Wuhan	N/A	Tujialing Raw Fair	open-air in normal temperature	Frozen
502	WHJ983	frozen beef	Wuhan	Wuhan	N/A	Tujialing Raw Fair	open-air in normal temperature	Frozen
503	WHN997	frozen broiler wing	Wuhan	Wuhan	20121009	20121009	open-air in normal temperature	Frozen
504	WHN998	frozen broiler leg	Wuhan	Wuhan	N/A	Yushun meat market	open-air in normal temperature	Frozen
505	CDC1016	dumpling	Chengdu	Zhengzhou	20120905	Yankou people's shopping malls	Frozen with sealed	Frozen
506	CDC1017	dumpling	Chengdu	Zhengzhou	20120922	Haolaiwu household mall	Frozen with sealed	Frozen
507	CDC1018	steamed bun stuffed	Chengdu	Sichuan	20120828	Haolaiwu household mall	Frozen with sealed	Frozen
508	CDJ1032	frozen broiler leg	Chengdu	Chengdu	20121016	farmer's markets	Frozen in bulk	Frozen
509	CDJ1033	frozen beef	Chengdu	Chengdu	20121016	Bluestone bridge market	Frozen in bulk	Frozen
510	CDN1047	frozen broiler wing	Chengdu	Chengdu	N/A	Randeng market	Frozen in bulk	Frozen
511	CDN1048	frozen broiler leg	Chengdu	Chengdu	N/A	Randeng market	Frozen in bulk	Frozen
512	KMC1066	dumpling	Kunming	Guizhou Guiyang	2012.08.25	Carrefour	Frozen with sealed	N/A
513	KMC1067	dumpling	Kunming	Zhejiang Jiaxing	2012.09.14	Wal-Mart	Frozen with sealed	N/A
514	KMC1068	steamed bun stuffed	Kunming	Tianjin Xinqing	2012.06.02	Wal-Mart	Frozen with sealed	N/A
515	KMJ1082	frozen broiler leg	Kunming	Kunming	2012.11.04	Donghua comprehensive market	Frozen in bulk	N/A
516	KMJ1083	Frozen beef	Kunming	Kunming	2012.11.04	Donghua comprehensive market	Frozen in bulk	N/A
517	KMN1097	frozen broiler wing	Kunming	Kunming	2012.11.04	Donghua comprehensive market	Frozen in bulk	N/A
518	KMN1098	frozen ham	Kunming	Kunming	2012.11.04	Donghua comprehensive market	Frozen in bulk	N/A
519	LZC1116	dumpling	Lanzhou	Zhengzhou	20121007	Lanzhou Hualian Supermarket	Frozen in bulk	Frozen
520	LZC1117	dumpling	Lanzhou	Chengdu	20121030	China Resources Vanguard	Frozen in bulk	Frozen
521	LZC1118	Vegetarian Bun	Lanzhou	Chengdu	20121012	Lanzhou Hualian Supermarket	Frozen in bulk	Frozen
522	LZJ1132	frozen broiler leg	Lanzhou	Liaoning	N/A	Zhangye Road market	cold storage in bulk	Frozen
523	LZJ1133	mutton	Lanzhou	Lanzhou	N/A	Zhangye Road market	Frozen with sealed	Frozen
524	LZN1147	frozen broiler wing	Lanzhou	N/A	N/A	Bulan road street market	cold storage in bulk	Frozen
525	LZN1148	frozen broiler leg	Lanzhou	N/A	N/A	Bulan road street market	cold storage in bulk	Frozen
526	HEBC1166	dumpling	Haerbin	Haerbin	2012.10.26	Wal-Mart	Frozen with sealed	N/A
527	HEBC1167	dumpling	Haerbin	Haerbin	2012.10.19	Zhongyanghong supermarket	Frozen with sealed	N/A

528	HEBC1168	steamed stuffed bun	Haerbin	Haerbin	2012.10.05	Zhongyanghong supermarket	Frozen with sealed	N/A
529	HEBJ1182	frozen broiler leg	Haerbin	Haerbin	N/A	Hada market	Frozen in bulk	N/A
530	HEBJ1183	mutton	Haerbin	Haerbin	2012.11.20	Hada market	Frozen in bulk	N/A
531	HEBN1197	frozen broiler wing	Haerbin	Haerbin	N/A	Daoli market	Frozen in bulk	N/A
532	HEBN1198	mutton	Haerbin	Haerbin	N/A	Hada market	Frozen in bulk	N/A
533	XAC1216	dumpling	Xi'an	Xi'an	2012.11.29	China Resources Vanguard	packaging in freezer	N/A
534	XAC1217	dumpling	Xi'an	Xi'an	2012.11.29	Wal-Mart	packagingin freezer	N/A
535	XAC1218	Baozi	Xi'an	Xi'an	2012.11.29	China Resources Vanguard	packagingin freezer	N/A
536	XAJ1232	frozen broiler leg	Xi'an	Xi'an	2012.11.29	Xianxin aquatic market	freezer in bulk	N/A
537	XAJ1233	mutton	Xi'an	Xi'an	2012.11.29	Guanghuixiang Fair	freezer in bulk	N/A
538	XAN1247	frozen broiler wing	Xi'an	Xi'an	2012.11.29	Wangjiacun Fair	freezer in bulk	N/A
539	XAN1248	Frozen duck leg	Xi'an	Xi'an	2012.11.29	Wangjiacun Fair	freezer in bulk	N/A
540	TYC1266	dumpling	Taiyuan	Liaoning Chaoyang	20120925	Meitehao supermarket	Frozen with sealed	N/A
541	TYC1267	dumpling	Taiyuan	Zhengzhou	20120914	Wal-Mart	Frozen with sealed	N/A
542	TYC1268	steamed bun stuffed	Taiyuan	Zhengzhou	20121012	Meitehao supermarket	Frozen with sealed	N/A
543	TYJ1282	frozen broiler leg	Taiyuan	Taiyuan	N/A	Haomeite yifen Fair	Frozen in bulk	N/A
544	TYJ1283	mutton	Taiyuan	Taiyuan	N/A	Upwell Supermarket	Frozen in bulk	N/A
545	TYN1297	frozen broiler wing	Taiyuan	Taiyuan	N/A	Taiyuan fruit north market	Frozen in bulk	N/A
546	TYN1298	frozen broiler leg	Taiyuan	Taiyuan	N/A	Taiyuan fruit north market	Frozen in bulk	N/A
547	BJC1316	dumpling	Beijing	Beijing	12.12.01	Carrefour	Frozen with sealed	N/A
548	BJC1317	Chicken dumpling	Beijing	N/A	12.12.09	Wal-Mart	Frozen with sealed	N/A
549	BJC1318	Steamed Bun	Beijing	Tianjin	12.12.13	Carrefour	Frozen with sealed	N/A
550	BJJ1332	frozen broiler leg	Beijing	N/A	12.12.18	Nongguangli Fair	Frozen in bulk	N/A
551	BJJ1333	frozen beef	Beijing	N/A	N/A	Nongguangli Fair	Frozen with box-packed	N/A
552	BJN1347	frozen broiler wing	Beijing	N/A	N/A	Nongguangli Fair	Frozen in bulk	N/A
553	BJN1348	frozen broiler leg	Beijing	N/A	N/A	Nongguangli Fair	Frozen in bulk	N/A
554	JNC1366	dumpling	Jinan	Henan Shangqiu	20121126	Wal-Mart	Frozen with sealed	Frozen
555	JNC1367	dumpling	Jinan	Henan Shangqiu	20121121	TESCO	Frozen with sealed	Frozen
556	JNC1368	dumpling	Jinan	Shandong Taian	20121123	Wal-Mart	Frozen with sealed	Frozen
557	JNJ1382	frozen broiler leg	Jinan	N/A	N/A	Minghu market	cold storage in bulk	Frozen
558	JNJ1383	frozen mutton	Jinan	N/A	N/A	Weiyi road Fair	Frozen with sealed	Frozen
559	JNN1397	frozen broiler wing	Jinan	N/A	N/A	Minghu market	cold storage in bulk	Frozen
560	JNN1398	frozen broiler leg	Jinan	N/A	N/A	Minghu market	cold storage in bulk	Frozen
561	YXC1416	dumpling	Guangzhou	Linfen	20121119	Tesco	Frozen with sealed	Frozen
562	YXC1417	steamed bun stuffed	Guangzhou	Shenzhen	20121215	Wal-market	Frozen with sealed	Frozen
563	YXC1418	dumpling	Guangzhou	Foshan	20130226	Tesco	Frozen with sealed	Frozen
564	YXJ1442	frozen broiler wing	Guangzhou	N/A	N/A	Huifu new street Fair	Frozen in bulk	Frozen
565	YXJ1443	frozen broiler leg	Guangzhou	N/A	N/A	Shaheding market	Frozen in bulk	Frozen
566	YXJ1444	pork chop	Guangzhou	N/A	N/A	Huifu new street Fair	Frozen with sealed	Frozen
567	YXJ1445	frozen mutton	Guangzhou	N/A	N/A	Shaheding market	Frozen with sealed	Frozen
568	LWC1466	dumpling	Guangzhou	N/A	N/A	Carrefour	packing in freezer	N/A

569		LWC1467	dumpling	Guangzhou	N/A	20121221	Parknshop	packing in freezer	N/A
570		LWC1468	dumpling	Guangzhou	N/A		Carrefour	packing in freezer	N/A
571		LWN1492	frozen broiler leg	Guangzhou	Guangzhou	N/A	Zilai meat market	Frozen in bulk	N/A
572		LWN1493	frozen broiler leg	Guangzhou	Guangzhou	20130312	Longjin meat Market	Frozen in bulk	N/A
573		LWN1494	frozen broiler wing	Guangzhou	Guangzhou	N/A	Zilai meat market	Frozen in bulk	N/A
574	**Quick-frozen food**	LWN1495	mutton	Guangzhou	Guangzhou	20130312	Longjin meat Market	packing in freezer	N/A
575		PYC1516	Steamed Bun Stuffed	Guangzhou	N/A	N/A	Parknshop	packing in freezer	N/A
576		PYC1517	dumpling	Guangzhou	N/A	N/A	China Resources Vanguard	packing in freezer	N/A
577		PYC1518	Steamed Bun Stuffed	Guangzhou	N/A	N/A	Parknshop	packing in freezer	N/A
578		PYN1542	frozen broiler leg	Guangzhou	Guangzhou	N/A	Rainbow meat vegetable market	Frozen in bulk	N/A
579		PYN1543	frozen broiler leg	Guangzhou	Guangzhou	N/A	Jushu meat vegetable market	Frozen in bulk	N/A
580		PYN1544	frozen broiler wing	Guangzhou	Guangzhou	N/A	Rainbow meat vegetable market	Frozen in bulk	N/A
581		PYN1545	mutton	Guangzhou	Guangzhou	N/A	Jushu meat vegetable market	Frozen with sealed	N/A
582		CHC1566	dumpling	Guangzhou	N/A	N/A	Yonghenglong west street supermarket	Frozen with sealed	Frozen
583		CHC1567	dumpling	Guangzhou	Guangzhou	13.02.01	Wanhe shopping mall	Frozen with sealed	Frozen
584		CHC1568	minced pork bun	Guangzhou	N/A	N/A	Yonghenglong west street supermarket	Frozen with sealed	Frozen
585		CHJ1592	frozen broiler wing	Guangzhou	N/A	N/A	west street market	Frozen in bulk	Frozen
586		CHJ1593	frozen broiler leg	Guangzhou	N/A	N/A	Hedong meat vegetable market	Frozen in bulk	Frozen
587		CHJ1594	Frozen meat	Guangzhou	N/A	N/A	west street market	Frozen in bulk	Frozen
588		CHJ1595	mutton	Guangzhou	N/A	N/A	Hedong meat vegetable market	Frozen with sealed	Frozen
589		ZCC1616	dumpling	Guangzhou	Chengdu	13.01.31	RT-Mart	packing in freezer	Frozen
590		ZCC1617	meat dumpling	Guangzhou	Shenzhen	2013.1.31	Renrenle Zengcheng shopping mall	Frozen with sealed	Frozen
591		ZCC1618	Baozi	Guangzhou	Shenzhen	N/A	RT-Mart	packing in freezer	Frozen
592		ZCJ1642	frozen broiler wing	Guangzhou	N/A	N/A	Licheng east Fair	Frozen in bulk	Frozen
593		ZCJ1643	frozen broiler wing	Guangzhou	N/A	N/A	Xiajie Fair	Frozen in bulk	Frozen
594		ZCJ1644	Frozen meat	Guangzhou	N/A	N/A	Licheng east Fair	Frozen in bulk	Frozen
595		ZCJ1645	Frozen Chicken	Guangzhou	N/A	N/A	Xiajie Fair	Frozen in bulk	Frozen
596		SZC1666	dumpling	Shenzhen	Guangzhou	2013.3.5	Xinyijia	packing in freezer	Frozen
597		SZC1667	dumpling	Shenzhen	Henan Zhengzhou	2013.3.26	China Resources Vanguard	packing in freezer	Frozen
598		SZC1668	mutton dumpling	Shenzhen	Shenzhen	2013.3.6	Xinyijia	packing in freezer	Frozen
599		SZJ1692	frozen broiler wing	Shenzhen	N/A	N/A	Mingli market	Frozen in bulk	Frozen
600		SZJ1693	frozen broiler leg	Shenzhen	N/A	N/A	Mingli market	Frozen in bulk	Frozen
601		SZJ1694	meat	Shenzhen	N/A	N/A	Hubei east market	quick-frozen	Frozen
602		SZJ1695	frozen broiler wing	Shenzhen	N/A	N/A	Hubei east market	Frozen in bulk	Frozen
603		STC1716	steamed stuffed bun	Shantou	Guangzhou	2013.5.6	Lotus	packing in freezer	Frozen
604		STC1717	steamed bun stuffed	Shantou	Henan Zhengzhou	2013.3.31	Lotus	packing in freezer	Frozen
605		STC1718	dumpling	Shantou	N/A	N/A	Wal-Mart	packing in freezer	Frozen
606		STJ1742	frozen broiler wing	Shantou	N/A	N/A	Donghu street market	Frozen in bulk	Frozen
607		STJ1743	frozen broiler leg	Shantou	N/A	N/A	Lotus	Frozen in bulk	Frozen
608		STJ1744	frozen broiler leg	Shantou	N/A	N/A	Lotus	Frozen in bulk	Frozen
609		STJ1745	frozen mutton	Shantou	N/A	N/A	Wal-Mart	Frozen in bulk	Frozen

610	ZJC1766	dumpling	Zhanjiang	Zhengshi	20130326	Aihua supermarket	packing in freezer	Frozen
611	ZJC1767	dumpling	Zhanjiang	Shantou	20130423	Wal-Mart	packing in freezer	Frozen
612	ZJC1768	mutton dumpling	Zhanjiang	Zhanjiang	20121207	Aihua supermarket	packing in freezer	Frozen
613	ZJJ1792	frozen broiler wing	Zhanjiang	Zhanjiang	20130526	Cikan Nanhua market	Frozen in bulk	Frozen
614	ZJJ1793	frozen broiler wing	Zhanjiang	Zhanjiang	20130526	Cikan north bridge market	Frozen in bulk	Frozen
615	ZJJ1794	frozen broiler leg	Zhanjiang	Zhanjiang	20130526	Cikan Nanhua market	Frozen in bulk	Frozen
616	ZJJ1795	frozen broiler leg	Zhanjiang	Zhanjiang	20130526	Cikan north bridge market	Frozen in bulk	Frozen
617	SGC1816	dumpling	Shaoguan	N/A	N/A	RT-Mart	Frozen with sealed	Frozen
618	SGC1817	dumpling	Shaoguan	N/A	N/A	Wal-Mart	Frozen with sealed	Frozen
619	SGC1818	dumpling	Shaoguan	N/A	N/A	RT-Mart	Frozen with sealed	Frozen
620	SGJ1842	frozen broiler wing	Shaoguan	N/A	N/A	Xinglong market	Frozen in bulk	Frozen
621	SGJ1843	frozen broiler leg	Shaoguan	N/A	N/A	Fengcai market	Frozen in bulk	Frozen
622	SGJ1844	frozen broiler leg	Shaoguan	N/A	N/A	Xinglong market	Frozen in bulk	Frozen
623	SGJ1845	frozen broiler wing	Shaoguan	N/A	N/A	Wal-Mart	Frozen in bulk	Frozen
624	HYC1866	steamed bun stuffed	Heyuan	N/A	N/A	Renrenle supermarket	Frozen in bulk	Frozen
625	HYC1867	dumpling	Heyuan	N/A	N/A	Guangsheng market	Frozen in bulk	Frozen meat stuffing
626	HYC1868	dumpling	Heyuan	N/A	N/A	Renrenle supermarket	Frozen in bulk	Frozen meat stuffing
627	HYJ1892	frozen broiler wing	Heyuan	N/A	N/A	Yuancheng center market	Frozen in bulk	Frozen
628	HYJ1893	frozen broiler leg	Heyuan	N/A	N/A	Xingyuan market	Frozen in bulk	Frozen
629	HYJ1894	frozen broiler leg	Heyuan	N/A	N/A	Yuancheng center market	Frozen in bulk	Frozen
630	HYJ1895	frozen broiler wing	Heyuan	N/A	N/A	Xingyuan market	Frozen in bulk	Frozen
631	FZC2216	dumpling	Fuzhou	N/A	N/A	Wal-Mart	in freezer	N/A
632	FZC2217	dumpling	Fuzhou	N/A	N/A	Carrefour	in freezer	N/A
633	FZC2218	Baozi	Fuzhou	N/A	N/A	Wal-Mart	in freezer	N/A
634	FZJ2242	frozen broiler wing	Fuzhou	N/A	N/A	Xiyingli market	in freezer	Frozen
635	FZJ2243	frozen chicken paw	Fuzhou	N/A	N/A	Xiyingli market	in freezer	Frozen
636	FZJ2244	frozen meat	Fuzhou	N/A	N/A	Xiyingli market	in freezer	Frozen
637	FZJ2245	Frozen mutton	Fuzhou	N/A	N/A	Xiyingli market	in freezer	Frozen
638	NNC2266	dumpling	Nanning	Nanning	N/A	Wal-Mart	Frozen in bulk	Frozen
639	NNC2267	dumpling	Nanning	Nanning	N/A	China Resources Vanguard	Frozen in bulk	Frozen
640	NNC2268	steamed bun stuffed	Nanning	Nanning	N/A	Wal-Mart	Frozen in bulk	Frozen
641	NNJ2292	frozen broiler wing	Nanning	N/A	N/A	Dancun market	Frozen in bulk	Frozen
642	NNJ2293	frozen broiler leg	Nanning	N/A	N/A	Dancun market	Frozen in bulk	Frozen
643	NNJ2294	frozen chicken paw	Nanning	N/A	N/A	Dancun market	Frozen in bulk	Frozen
644	NNJ2295	Frozen mutton	Nanning	Heilongjiang		China Resources Vanguard	Frozen in bulk	Frozen
645	XMC2316	dumpling	Xiamen	Henan	13.08.09	Tesco	Frozen in bulk	Frozen
646	XMC2317	dumpling	Xiamen	Wuxi	13.11.03	Wal-Mart	Frozen in bulk	Frozen
647	XMC2318	steamed bun stuffed	Xiamen	Henan Zhengzhou	13.09.09	Tesco	Frozen in bulk	Frozen
648	XMJ2342	frozen broiler wing	Xiamen	N/A	N/A	The eight market	Frozen in bulk	Frozen
649	XMJ2343	frozen broiler leg	Xiamen	N/A	N/A	Ruijing market	Frozen in bulk	Frozen
650	XMJ2344	Frozen muttonfrozen chicken paw	Xiamen	N/A	N/A	The eight market	Frozen in bulk	Frozen

651		XMJ2345	Frozen muttonfrozen chicken paw	Xiamen	N/A	N/A	Ruijing market	Frozen in bulk	Frozen
652		BHC2366	dumpling	Beihai	Henan Zhengzhou	13.10.29	Hean supermarket	Frozen in bulk	frozen
653		BHC2367	dumpling	Beihai	Guangxi Nanning	13.11.28	RT-Mart	Frozen in bulk	frozen
654		BHC2368	steamed stuffed bun	Beihai	Guangxi Nanning	13.09.27	Hean supermarket	Frozen in bulk	frozen
655		BHJ2392	frozen broiler wing	Beihai	N/A	N/A	Guizhou road market	Frozen in bulk	frozen
656		BHJ2393	frozen broiler leg	Beihai	N/A	N/A	Beijing road market	Frozen in bulk	frozen
657		BHJ2394	frozen broiler wing	Beihai	N/A	N/A	Guizhou road market	Frozen in bulk	frozen
658		BHJ2395	Frozen sliced mutton	Beihai	N/A	N/A	RT-Mart	Frozen in bulk	frozen
659		HKC2416	dumpling	Haikou	Gujian Fuzhou	13.10.17	Carrefour	Frozen in bulk	Frozen
660		HKC2417	steamed bun stuffed	Haikou	Gujian Fuzhou	13.11.26	Parknshop	Frozen in bulk	Frozen
661		HKC2418	steamed stuffed bun	Haikou	Guangdong Foshan	13.11.04	Carrefour	Frozen in bulk	Frozen
662		HKJ2442	frozen broiler wing	Haikou	N/A	N/A	Longshepo market	Frozen in bulk	Frozen
663		HKJ2443	Frozen muttonfrozen chicken paw	Haikou	N/A	N/A	Mingsheng market	Frozen in bulk	Frozen
664		HKJ2444	Frozen muttonfrozen chicken paw	Haikou	N/A	N/A	Longshepo market	Frozen in bulk	Frozen
665		HKJ2445	mutton	Haikou	Fujian Fuzhou	13.09.25	Parknshop	Frozen in bulk	Frozen
666		SYC2466	dumpling	Sanya	guangzhou	20131120	Fuleduo supermarket	packing in freezer	N/A
667		SYC2467	dumpling	Sanya	Zhengzhou	20131223	Haowang supermarket	packing in freezer	N/A
668		SYC2468	steamed bun stuffed	Sanya	Zhengzhou	20131208	Fuleduo supermarket	packing in freezer	N/A
669		SYJ2492	frozen broiler wing	Sanya	N/A	N/A	The one market	in bulk	N/A
670		SYJ2493	frozen broiler leg	Sanya	N/A	N/A	City center market	in bulk	N/A
671		SYJ2494	Frozen Chicken	Sanya	N/A	N/A	The one market	in bulk	N/A
672		SYJ2495	Quick-frozen Lamb Rolls	Sanya	N/A	N/A	City center market	in bulk	N/A
673		SHC819	flammulina velutipes	Shanghai	N/A	2012.09.05	Carrefour	packaging in law temperature	Packing
674		SHC820	hypsizigus marmoreus	Shanghai	N/A	2012.09.05	Carrefour	packaging in law temperature	Packing
675		SHC821	lentinus edodes	Shanghai	N/A	2012.09.05	Carrefour	packaging in law temperature	Packing
676		SHJ834	flammulina velutipes	Shanghai	N/A	2012.09.05	Liuli Fair	normal temperature in bulk	N/A
677		SHJ835	pleurotus eryngii	Shanghai	N/A	2012.09.05	Liuli Fair	normal temperature in bulk	N/A
678		SHJ836	Pleurotus ostreatus	Shanghai	N/A	2012.09.05	Liuli Fair	normal temperature in bulk	N/A
679		SHN849	flammulina velutipes	Shanghai	N/A	2012.09.05	Shanghai Yunlian market	normal temperature in bulk	N/A
680		SHN850	Volvariella volvacea	Shanghai	N/A	2012.09.05	Shanghai Yunlian market	normal temperature in bulk	N/A
681		HFC869	flammulina velutipes	Hefei	N/A	N/A	Carrefour	normal temperature in bulk	N/A
682		HFC870	pleurotus geesteranus	Hefei	N/A	N/A	Carrefour	normal temperature in bulk	N/A
683		HFC871	lentinus edodes	Hefei	N/A	N/A	Carrefour	normal temperature in bulk	N/A
684		HFJ884	flammulina velutipes	Hefei	N/A	N/A	Zhong market	normal temperature in bulk	N/A
685		HFJ885	pleurotus eryngii	Hefei	N/A	N/A	Zhong market	normal temperature in bulk	N/A
686		HFJ886	Pleurotus ostreatus	Hefei	N/A	N/A	Zhong market	normal temperature in bulk	N/A
687		HFJ899	flammulina velutipes	Hefei	N/A	N/A	Xinghua market	normal temperature in bulk	N/A
688		HFJ900	hypsizigus marmoreus	Hefei	N/A	N/A	Xinghua market	normal temperature in bulk	N/A
689		NCC919	flammulina velutipes	Nanchang	Nanchang	N/A	Wal-Mart	packaging in normal temperature	Fresh
690		NCC920	hypsizigus marmoreus	Nanchang	Nanchang	N/A	RT-Mart	packaging in normal temperature	Fresh
691		NCC921	lentinus edodes	Nanchang	Nanchang	N/A	Wal-Mart	packaging in normal temperature	Fresh

692	NCJ934	flammulina velutipes	Nanchang	Nanchang	N/A	Dunzitang Fair	normal temperature in bulk	Fresh
693	NCJ935	pleurotus eryngii	Nanchang	Nanchang	N/A	Fushan road Fair	normal temperature in bulk	Fresh
694	NCJ936	Pleurotus ostreatus	Nanchang	Nanchang	N/A	Dunzitang Fair	normal temperature in bulk	Fresh
695	NCN949	flammulina velutipes	Nanchang	Nanchang	N/A	Xi'mazhuang Fair	normal temperature in bulk	Fresh
696	NCN950	Pleurotus ostreatus	Nanchang	Nanchang	N/A	Xi'mazhuang Fair	normal temperature in bulk	Fresh
697	WHC969	flammulina velutipes	Wuhan	Hubei	20121009	Lotus	ox-packed in normal temperatu	Fresh
698	WHC970	hypsizigus marmoreus	Wuhan	Hubei	20121009	Wal-Mart	ox-packed in normal temperatu	Fresh
699	WHC971	lentinus edodes	Wuhan	Hubei	20121009	Wal-Mart	ox-packed in normal temperatu	Fresh
700	WHJ984	flammulina velutipes	Wuhan	Wuhan	20121009	Tujialing Raw Fair	open-air in normal temperature	Fresh
701	WHJ985	pleurotus eryngii	Wuhan	Wuhan	20121009	Tujialing Raw Fair	open-air in normal temperature	Fresh
702	WHJ986	Pleurotus ostreatus	Wuhan	Wuhan	20121009	Tujialing Raw Fair	open-air in normal temperature	Fresh
703	WHN999	flammulina velutipes	Wuhan	Wuhan	20121009	Yushun meat market	open-air in normal temperature	Fresh
704	WHN1000	Pleurotus ostreatus	Wuhan	Wuhan	20121009	Yushun meat market	open-air in normal temperature	Fresh
705	CDC1019	flammulina velutipes	Chengdu	Chengdu	20121016	Haolaiwu household mall	packaging in normal temperature	fresh
706	CDC1020	hypsizigus marmoreus	Chengdu	Chengdu	20121016	Haolaiwu household mall	packaging in normal temperature	fresh
707	CDC1021	lentinus edodes	Chengdu	Chengdu	20121016	Yankou people's shopping malls	packaging in normal temperature	fresh
708	CDJ1034	flammulina velutipes	Chengdu	Chengdu	20121016	Bluestone bridge market	normal temperature in bulk	fresh
709	CDJ1035	lentinus edodes	Chengdu	Chengdu	20121016	Bluestone bridge market	normal temperature in bulk	fresh
710	CDJ1036	Pleurotus ostreatus	Chengdu	Chengdu	20121016	Bluestone bridge market	normal temperature in bulk	fresh
711	CDN1049	flammulina velutipes	Chengdu	Chengdu	N/A	Xiaojiahe market	normal temperature in bulk	fresh
712	CDN1050	Pleurotus ostreatus	Chengdu	Chengdu	N/A	Xiaojiahe market	normal temperature in bulk	fresh
713	KMC1069	flammulina velutipes	Kunming	Kunming	2012.11.04	Carrefour	packaging in normal temperature	N/A
714	KMC1070	hypsizigus marmoreus	Kunming	Kunming	2012.11.04	Carrefour	packaging in normal temperature	N/A
715	KMC1071	lentinus edodes	Kunming	Kunming	2012.11.04	Wal-Mart	packaging in normal temperature	N/A
716	KMJ1084	flammulina velutipes	Kunming	Kunming	2012.11.04	Donghua comprehensive market	normal temperature in bulk	N/A
717	KMJ1085	Mushroom	Kunming	Kunming	2012.11.04	Tianyuanli yintan farmer's markets	normal temperature in bulk	N/A
718	KMJ1086	pleurotus eryngii	Kunming	Kunming	2012.11.04	Tianyuanli yintan farmer's markets	normal temperature in bulk	N/A
719	KMN1099	flammulina velutipes	Kunming	Kunming	2012.11.04	Tianyuanli yintan farmer's markets	normal temperature in bulk	N/A
720	KMN1100	Pleurotus ostreatus	Kunming	Kunming	2012.11.04	Donghua comprehensive market	normal temperature in bulk	N/A
721	LZC1119	flammulina velutipes	Lanzhou	N/A	N/A	China Resources Vanguard	packaging in normal temperature	Fresh
722	LZC1120	hypsizigus marmoreus	Lanzhou	N/A	N/A	Lanzhou Hualian Supermarket	packaging in normal temperature	Fresh
723	LZC1121	lentinus edodes	Lanzhou	N/A	N/A	China Resources Vanguard	packaging in normal temperature	Fresh
724	LZJ1134	flammulina velutipes	Lanzhou	N/A	N/A	Zhangye Road market	lowl temperature in bulk	Fresh
725	LZJ1135	pleurotus eryngii	Lanzhou	N/A	N/A	Zhangye Road market	lowl temperature in bulk	Fresh
726	LZJ1136	agaricus bisporus	Lanzhou	N/A	N/A	Zhangye Road market	lowl temperature in bulk	Fresh
727	LZN1149	flammulina velutipes	Lanzhou	N/A	N/A	Bulan road street market	lowl temperature in bulk	Fresh
728	LZN1150	Pleurotus ostreatus	Lanzhou	N/A	N/A	Bulan road street market	lowl temperature in bulk	Fresh
729	HEBC1169	flammulina velutipes	Haerbin	N/A	N/A	Wal-Mart	packaging in normal temperature	N/A
730	HEBC1170	pleurotus eryngii	Haerbin	N/A	N/A	Wal-Mart	packaging in normal temperature	N/A
731	HEBC1171	lentinus edodes	Haerbin	N/A	N/A	Wal-Mart	packaging in normal temperature	N/A
732	HEBN1184	flammulina velutipes	Haerbin	N/A	N/A	Hada market	normal temperature in bulk	N/A

733	HEBN1185	pleurotus eryngii	Haerbin	N/A	N/A	Hada market	normal temperature in bulk	N/A
734	HEBN1186	hypsizigus marmoreus	Haerbin	N/A	N/A	Hada market	normal temperature in bulk	N/A
735	HEBN1199	flammulina velutipes	Haerbin	N/A	N/A	Daoli market	normal temperature in bulk	N/A
736	HEBN1200	Pleurotus ostreatus	Haerbin	N/A	N/A	Daoli market	normal temperature in bulk	N/A
737	XAC1219	flammulina velutipes	Xi'an	Xi'an	2012.11.29	China Resources Vanguard	normal temperature in bulk	N/A
738	XAC1220	hypsizigus marmoreus	Xi'an	Xi'an	2012.11.29	Wal-Mart	normal temperature in bulk	N/A
739	XAC1221	lentinus edodes	Xi'an	Xi'an	2012.11.29	China Resources Vanguard	normal temperature in bulk	N/A
740	XAJ1234	flammulina velutipes	Xi'an	Xi'an	2012.11.29	Guanghuixiang Fair	normal temperature in bulk	N/A
741	XAJ1235	pleurotus eryngii	Xi'an	Xi'an	2012.11.29	Guanghuixiang Fair	normal temperature in bulk	N/A
742	XAJ1236	cordycepin flower	Xi'an	Xi'an	2012.11.29	Xianxin aquatic market	normal temperature in bulk	N/A
743	XAN1249	flammulina velutipes	Xi'an	Xi'an	2012.11.29	Wangjiacun Fair	normal temperature in bulk	N/A
744	XAN1250	Pleurotus ostreatus	Xi'an	Xi'an	2012.11.29	Sankeshu Fair	normal temperature in bulk	N/A
745	TYC1269	flammulina velutipes	Taiyuan	Taiyuan	20121211	Meitehao supermarket	packaging in normal temperature	N/A
746	TYC1270	hypsizigus marmoreus	Taiyuan	Taiyuan	20121211	Meitehao supermarket	packaging in normal temperature	N/A
747	TYC1271	lentinus edodes	Taiyuan	Taiyuan	20121211	Wal-Mart	packaging in normal temperature	N/A
748	TYJ1284	flammulina velutipes	Taiyuan	Taiyuan	20121211	Upwell Supermarket	normal temperature in bulk	N/A
749	TYJ1285	pleurotus eryngii	Taiyuan	Taiyuan	20121211	Upwell Supermarket	normal temperature in bulk	N/A
750	TYJ1286	Pleurotus ostreatus	Taiyuan	Taiyuan	20121211	Upwell Supermarket	normal temperature in bulk	N/A
751	TYN1299	flammulina velutipes	Taiyuan	Taiyuan	20121211	Haomeite yifen Fair	normal temperature in bulk	N/A
752	TYN1300	Pleurotus ostreatus	Taiyuan	Taiyuan	20121211	Haomeite yifen Fair	normal temperature in bulk	N/A
753	BJC1319	flammulina velutipes	Beijing	N/A	12.12.18	Wal-Mart	packaging in normal temperature	N/A
754	BJC1320	hypsizigus marmoreus	Beijing	N/A	12.12.18	Carrefour	normal temperature in bulk	N/A
755	BJC1321	lentinus edodes	Beijing	N/A	12.12.18	Wal-Mart	normal temperature in bulk	N/A
756	BJJ1334	flammulina velutipes	Beijing	N/A	12.12.18	Nongguangli Fair	normal temperature in bulk	N/A
757	BJJ1335	pleurotus eryngii	Beijing	N/A	12.12.18	Nongguangli Fair	normal temperature in bulk	N/A
758	BJJ1336	Pleurotus ostreatus	Beijing	N/A	12.12.18	Nongguangli Fair	normal temperature in bulk	N/A
759	BJN1349	flammulina velutipes	Beijing	N/A	N/A	Dingsheng market	normal temperature in bulk	N/A
760	BJN1350	Pleurotus ostreatus	Beijing	N/A	N/A	Dingsheng market	normal temperature in bulk	N/A
761	JNC1369	flammulina velutipes	Jinan	N/A	N/A	TESCO	packaging in normal temperature	Fresh
762	JNC1370	hypsizigus marmoreus	Jinan	N/A	N/A	Wal-Mart	packaging in normal temperature	Fresh
763	JNC1371	lentinus edodes	Jinan	N/A	N/A	TESCO	packaging in normal temperature	Fresh
764	JNJ1384	flammulina velutipes	Jinan	N/A	jiangsu	Weiyi road Fair	cold storage in bulk	Fresh
765	JNJ1385	lentinus edodes	Jinan	N/A	N/A	Weiyi road Fair	cold storage in bulk	Fresh
766	JNJ1386	white beech mushroom	Jinan	N/A	jiangsu	Weiyi road Fair	cold storage in bulk	Fresh
767	JNN1399	flammulina velutipes	Jinan	N/A	N/A	Weiyi road Fair	cold storage in bulk	Fresh
768	JNN1400	Pleurotus ostreatus	Jinan	N/A	N/A	Weiyi road Fair	cold storage in bulk	Fresh
769	YXC1419	flammulina velutipes	Guangzhou	N/A	N/A	Wal-market	packaging in low temperature	Fresh
770	YXC1420	hypsizigus marmoreus	Guangzhou	N/A	N/A	Tesco	packaging in low temperature	Fresh
771	YXC1421	lentinus edodes	Guangzhou	N/A	N/A	Wal-market	packaging in low temperature	Fresh
772	YXJ1446	flammulina velutipes	Guangzhou	Guangzhou	N/A	Huifu new street Fair	normal temperature in bulk	Fresh
773	YXJ1447	flammulina velutipes	Guangzhou	Guangzhou	N/A	Shaheding market	normal temperature in bulk	Fresh

774	Edible mushroom	YXJ1448	Pleurotus ostreatus	Guangzhou	Guangzhou	N/A	Huifu new street Fair	normal temperature in bulk	Fresh
775		YXJ1449	pleurotus eryngii	Guangzhou	Guangzhou	N/A	Huanghuagang meat market	normal temperature in bulk	Fresh
776		YXJ1450	Volvariella volvacea	Guangzhou	Guangzhou	N/A	Huifu new street Fair	normal temperature in bulk	Fresh
777		LWC1469	flammulina velutipes	Guangzhou	Guangzhou	20130312	Parknshop	normal temperature in bulk	Fresh
778		LWC1470	pleurotus geesteranus	Guangzhou	Guangzhou	20130312	Carrefour	normal temperature in bulk	Fresh
779		LWC1471	white beech mushroom	Guangzhou	Guangzhou	20130312	Parknshop	normal temperature in bulk	Fresh
780		LWN1496	flammulina velutipes	Guangzhou	Guangzhou	20130312	Zilai meat market	normal temperature in bulk	Fresh
781		LWN1497	flammulina velutipes	Guangzhou	Guangzhou	20130312	Longjin middle road Hongfu market	normal temperature in bulk	Fresh
782		LWN1498	brown mushroom	Guangzhou	Guangzhou	20130312	Zilai meat market	normal temperature in bulk	Fresh
783		LWN1499	pleurotus eryngii	Guangzhou	Guangzhou	20130312	Longjin middle road Hongfu market	normal temperature in bulk	Fresh
784		LWN1500	lentinus edodes	Guangzhou	Guangzhou	20130312	Carrefour	normal temperature in bulk	Fresh
785		PYC1519	flammulina velutipes	Guangzhou	Guangzhou	20130319	China Resources Vanguard	normal temperature in bulk	Fresh
786		PYC1520	flammulina velutipes	Guangzhou	Guangzhou	20130319	Parknshop	normal temperature in bulk	Fresh
787		PYC1521	white beech mushroom	Guangzhou	Guangzhou	20130319	China Resources Vanguard	normal temperature in bulk	Fresh
788		PYN1546	flammulina velutipes	Guangzhou	Guangzhou	20130319	Rainbow meat vegetable market	normal temperature in bulk	Fresh
789		PYN1547	flammulina velutipes	Guangzhou	Guangzhou	20130319	Jushu meat vegetable market	normal temperature in bulk	Fresh
790		PYN1548	brown mushroom	Guangzhou	Guangzhou	20130319	Rainbow meat vegetable market	normal temperature in bulk	Fresh
791		PYN1549	white beech mushroom	Guangzhou	Guangzhou	20130319	Jushu meat vegetable market	normal temperature in bulk	Fresh
792		PYN1550	hypsizigus marmoreus	Guangzhou	Guangzhou	20130319	Rainbow meat vegetable market	normal temperature in bulk	Fresh
793		CHC1569	flammulina velutipes	Guangzhou	N/A	N/A	Wanhe shopping mall	packaging in low temperature	fresh
794		CHC1570	hypsizigus marmoreus	Guangzhou	N/A	N/A	Yonghenglong west street supermarket	packaging in low temperature	fresh
795		CHC1571	lentinus edodes	Guangzhou	N/A	N/A	Wanhe shopping mall	packaging in low temperature	fresh
796		CHJ1596	flammulina velutipes	Guangzhou	N/A	N/A	west street market	normal temperature in bulk	fresh
797		CHJ1597	flammulina velutipes	Guangzhou	N/A	N/A	Hedong meat vegetable market	normal temperature in bulk	fresh
798		CHJ1598	Pleurotus ostreatus	Guangzhou	N/A	N/A	west street market	normal temperature in bulk	fresh
799		CHJ1599	pleurotus eryngii	Guangzhou	N/A	N/A	Hedong meat vegetable market	normal temperature in bulk	fresh
800		CHJ1600	Volvariella volvacea	Guangzhou	N/A	N/A	west street market	normal temperature in bulk	fresh
801		ZCC1619	flammulina velutipes	Guangzhou	N/A	N/A	Renrenle Zengcheng shopping mall	normal temperature in bulk	fresh
802		ZCC1620	hypsizigus marmoreus	Guangzhou	N/A	N/A	RT-Mart	normal temperature in bulk	fresh
803		ZCC1621	lentinus edodes	Guangzhou	N/A	N/A	Renrenle Zengcheng shopping mall	normal temperature in bulk	fresh
804		ZCJ1646	flammulina velutipes	Guangzhou	N/A	N/A	Licheng east Fair	normal temperature in bulk	N/A
805		ZCJ1647	flammulina velutipes	Guangzhou	N/A	N/A	Xiajie Fair	normal temperature in bulk	N/A
806		ZCJ1648	Pleurotus ostreatus	Guangzhou	N/A	N/A	Licheng east Fair	normal temperature in bulk	N/A
807		ZCJ1649	agaric	Guangzhou	N/A	N/A	Xiajie Fair	normal temperature in bulk	N/A
808		ZCJ1650	Volvariella volvacea	Guangzhou	N/A	N/A	Licheng east Fair	normal temperature in bulk	N/A
809		SZC1669	flammulina velutipes	Shenzhen	N/A	N/A	China Resources Vanguard	packaging in low temperature	N/A
810		SZC1670	coprinus comatus	Shenzhen	N/A	N/A	Xinyijia	packaging in low temperature	N/A
811		SZC1671	lentinus edodes	Shenzhen	N/A	N/A	China Resources Vanguard	packaging in low temperature	N/A
812		SZJ1696	flammulina velutipes	Shenzhen	N/A	N/A	Hubei east market	normal temperature in bulk	Fresh
813		SZJ1697	flammulina velutipes	Shenzhen	N/A	N/A	Mingli market	normal temperature in bulk	Fresh
814		SZJ1698	Pleurotus ostreatus	Shenzhen	N/A	N/A	Hubei east market	normal temperature in bulk	Fresh

815	SZJ1699	Pleurotus ostreatus	Shenzhen	N/A	N/A	Mingli market	normal temperature in bulk	Fresh
816	SZJ1600	Volvariella volvacea	Shenzhen	N/A	N/A	Hubei east market	normal temperature in bulk	Fresh
817	STC1719	agrocybe cylindracea	Shantou	N/A	N/A	Wal-Mart	packaging in low temperature	Fresh
818	STC1720	hypsizigus marmoreus	Shantou	N/A	N/A	Wal-Mart	packaging in low temperature	Fresh
819	STC1721	snake butter	Shantou	N/A	N/A	Wal-Mart	packaging in low temperature	Fresh
820	STJ1746	flammulina velutipes	Shantou	N/A	N/A	Donghu street market	normal temperature in bulk	Fresh
821	STJ1747	flammulina velutipes	Shantou	N/A	N/A	Longkou north road market	normal temperature in bulk	Fresh
822	STJ1748	Pleurotus ostreatus	Shantou	N/A	N/A	Donghu street market	normal temperature in bulk	Fresh
823	STJ1749	pleurotus geesteranus	Shantou	N/A	N/A	Longkou north road market	normal temperature in bulk	Fresh
824	STJ1750	Volvariella volvacea	Shantou	N/A	N/A	Longkou north road market	normal temperature in bulk	Fresh
825	ZJC1769	flammulina velutipes	Zhanjiang	Zhanjiang	20130525	Wal-Mart	packaging in low temperature	Fresh
826	ZJC1770	flammulina velutipes	Zhanjiang	Zhanjiang	20130526	Wal-Mart	packaging in low temperature	Fresh
827	ZJC1771	lentinus edodes	Zhanjiang	Zhanjiang	20130526	Wal-Mart	packaging in low temperature	Fresh
828	ZJJ1796	flammulina velutipes	Zhanjiang	Zhanjiang	20130526	Cikan Nanhua market	normal temperature in bulk	Fresh
829	ZJJ1797	flammulina velutipes	Zhanjiang	Zhanjiang	20130526	Cikan north bridge market	normal temperature in bulk	Fresh
830	ZJJ1798	Pleurotus ostreatus	Zhanjiang	Zhanjiang	20130526	Cikan Nanhua market	normal temperature in bulk	Fresh
831	ZJJ1799	Pleurotus ostreatus	Zhanjiang	Zhanjiang	20130526	Cikan north bridge market	normal temperature in bulk	Fresh
832	ZJJ1800	Volvariella volvacea	Zhanjiang	Zhanjiang	20130526	Cikan Nanhua market	normal temperature in bulk	Fresh
833	SGC1819	flammulina velutipes	Shaoguan	N/A	N/A	Wal-Mart	packaging in low temperature	fresh
834	SGC1820	pleurotus geesteranus	Shaoguan	N/A	N/A	RT-Mart	packaging in low temperature	fresh
835	SGC1821	lentinus edodes	Shaoguan	N/A	N/A	Wal-Mart	packaging in low temperature	fresh
836	SGJ1846	flammulina velutipes	Shaoguan	N/A	N/A	Xinglong market	normal temperature in bulk	fresh
837	SGJ1847	flammulina velutipes	Shaoguan	N/A	N/A	Fengcai market	normal temperature in bulk	fresh
838	SGJ1848	pleurotus eryngii	Shaoguan	N/A	N/A	Xinglong market	normal temperature in bulk	fresh
839	SGJ1849	Pleurotus ostreatus	Shaoguan	N/A	N/A	Fengcai market	normal temperature in bulk	fresh
840	SGJ1850	Volvariella volvacea	Shaoguan	N/A	N/A	Xinglong market	normal temperature in bulk	fresh
841	HYC1869	Volvariella volvacea	Heyuan	N/A	N/A	Guangsheng market	open-air in normal temperature	fresh
842	HYC1870	pleurotus geesteranus	Heyuan	N/A	N/A	Renrenle supermarket	open-air in normal temperature	fresh
843	HYC1871	white beech mushroom	Heyuan	N/A	N/A	Guangsheng market	open-air in normal temperature	fresh
844	HYJ1896	flammulina velutipes	Heyuan	N/A	N/A	Yuancheng center market	open-air in normal temperature	fresh
845	HYJ1897	flammulina velutipes	Heyuan	N/A	N/A	Xingyuan market	open-air in normal temperature	fresh
846	HYJ1898	pleurotus eryngii	Heyuan	N/A	N/A	Yuancheng center market	open-air in normal temperature	fresh
847	HYJ1899	Pleurotus ostreatus	Heyuan	N/A	N/A	Xingyuan market	open-air in normal temperature	fresh
848	HYJ1900	Volvariella volvacea	Heyuan	N/A	N/A	Yuancheng center market	open-air in normal temperature	fresh
849	FZC2219	flammulina velutipes	Fuzhou	N/A	N/A	Carrefour	packaging in normal temperatur	N/A
850	FZC2220	Hypsizygus marmoreus	Fuzhou	N/A	N/A	Wal-Mart	packaging in normal temperatur	N/A
851	FZC2221	lentinus edodes	Fuzhou	N/A	N/A	Carrefour	packaging in normal temperatur	N/A
852	FZJ2246	flammulina velutipes	Fuzhou	N/A	N/A	Xiyingli market	open-air in normal temperature	Fresh
853	FZJ2247	flammulina velutipes	Fuzhou	N/A	N/A	Xiyingli market	open-air in normal temperature	Fresh
854	FZJ2248	pleurotus eryngii	Fuzhou	N/A	N/A	Xiyingli market	open-air in normal temperature	Fresh
855	FZJ2249	Pleurotus ostreatus	Fuzhou	N/A	N/A	Xiyingli market	open-air in normal temperature	Fresh

856		FZJ2250	Volvariella volvacea	Fuzhou	N/A	N/A	Xiyingli market	open-air in normal temperature	Fresh
857		NNC2269	flammulina velutipes	Nanning	N/A	N/A	China Resources Vanguard	open-air in normal temperature	Fresh
858		NNC2270	hypsizigus marmoreus	Nanning	N/A	N/A	Wal-Mart	open-air in normal temperature	Fresh
859		NNC2271	lentinus edodes	Nanning	N/A	N/A	China Resources Vanguard	open-air in normal temperature	Fresh
860		NNJ2296	flammulina velutipes	Nanning	N/A	N/A	Dancun market	open-air in normal temperature	Fresh
861		NNJ2297	lentinus edodes	Nanning	N/A	N/A	Dancun market	open-air in normal temperature	Fresh
862		NNJ2298	pleurotus eryngii	Nanning	N/A	N/A	Dancun market	open-air in normal temperature	Fresh
863		NNJ2299	flammulina velutipes	Nanning	N/A	N/A	Dancun market	open-air in normal temperature	Fresh
864		NNJ2300	Volvariella volvacea	Nanning	N/A	N/A	Dancun market	open-air in normal temperature	Fresh
865		XMC2319	flammulina velutipes	Xiamen	N/A	N/A	Wal-Mart	open-air in normal temperature	fresh
866		XMC2320	hypsizigus marmoreus	Xiamen	N/A	N/A	Tesco	open-air in normal temperature	fresh
867		XMC2321	abalone mushroom	Xiamen	N/A	N/A	Wal-Mart	open-air in normal temperature	fresh
868		XMJ2346	flammulina velutipes	Xiamen	N/A	N/A	The eight market	open-air in normal temperature	fresh
869		XMJ2347	lentinus edodes	Xiamen	N/A	N/A	Ruijing market	open-air in normal temperature	fresh
870		XMJ2348	pleurotus eryngii	Xiamen	N/A	N/A	The eight market	open-air in normal temperature	fresh
871		XMJ2349	pleurotus eryngii	Xiamen	N/A	N/A	Ruijing market	open-air in normal temperature	fresh
872		XMJ2350	mushroom	Xiamen	N/A	N/A	The eight market	open-air in normal temperature	fresh
873		BHC2369	flammulina velutipes	Beihai	N/A	N/A	RT-Mart	open-air in normal temperature	fresh
874		BHC2370	white beech mushroom	Beihai	N/A	N/A	Hean supermarket	open-air in normal temperature	fresh
875		BHC2371	lentinus edodes	Beihai	N/A	N/A	RT-Mart	open-air in normal temperature	fresh
876		BHJ2396	flammulina velutipes	Beihai	N/A	N/A	Guizhou road market	open-air in normal temperature	fresh
877		BHJ2397	flammulina velutipes	Beihai	N/A	N/A	Beijing road market	open-air in normal temperature	fresh
878		BHJ2398	pleurotus eryngii	Beihai	N/A	N/A	Guizhou road market	open-air in normal temperature	fresh
879		BHJ2399	Pleurotus ostreatus	Beihai	N/A	N/A	Beijing road market	open-air in normal temperature	fresh
880		BHJ2400	Pleurotus ostreatus	Beihai	N/A	N/A	Guizhou road market	open-air in normal temperature	fresh
881		HKC2419	flammulina velutipes	Haikou	N/A	N/A	Parknshop	open-air in normal temperature	fresh
882		HKC2420	Hypsizygus marmoreus	Haikou	N/A	N/A	Carrefour	open-air in normal temperature	fresh
883		HKC2421	pleurotus eryngii	Haikou	N/A	N/A	Parknshop	open-air in normal temperature	fresh
884		HKJ2446	flammulina velutipes	Haikou	N/A	N/A	Longshepo market	open-air in normal temperature	fresh
885		HKJ2447	Pleurotus ostreatus	Haikou	N/A	N/A	Mingsheng market	open-air in normal temperature	fresh
886		HKJ2448	pleurotus eryngii	Haikou	N/A	N/A	Longshepo market	open-air in normal temperature	fresh
887		HKJ2449	flammulina velutipes	Haikou	N/A	N/A	Mingsheng market	open-air in normal temperature	fresh
888		HKJ2450	Pleurotus ostreatus	Haikou	N/A	N/A	Longshepo market	open-air in normal temperature	fresh
889		SYC2469	flammulina velutipes	Sanya	N/A	N/A	Haowang supermarket	packaging in normal temperature	N/A
890		SYC2470	Coprinus comatus	Sanya	N/A	N/A	Fuleduo supermarket	packaging in normal temperature	N/A
891		SYC2471	lentinus edodes	Sanya	N/A	N/A	Haowang supermarket	packaging in normal temperature	N/A
892		SYJ2496	flammulina velutipes	Sanya	N/A	N/A	The one market	open-air in normal temperature	N/A
893		SYJ2497	flammulina velutipes	Sanya	N/A	N/A	City center market	open-air in normal temperature	N/A
894		SYJ2498	pleurotus eryngii	Sanya	N/A	N/A	The one market	open-air in normal temperature	N/A
895		SYJ2499	Pleurotus ostreatus	Sanya	N/A	N/A	City center market	open-air in normal temperature	N/A
896		SYJ2500	lentinus edodes	Sanya	N/A	N/A	The one market	open-air in normal temperature	N/A

897		SHC813	lettuce	Shanghai	Shanghai	20120904	Carrefour	packaging in normal temperature	No water, no root
898		SHC814	caraway	Shanghai	N/A	N/A	Tesco	normal temperature in bulk	No water, no root
899		SHC815	Salad (mixed)	Shanghai	Shanghai	20120901	Carrefour	packaging in low temperature	Pre-cut
900		SHJ831	caraway	Shanghai	N/A	N/A	Pusan Road market	packaging in low temperature	No water, no root
901		SHN846	cucumber	Shanghai	N/A	N/A	Pusan road Fair	normal temperature in bulk	No water, no root
902		HFC863	caraway	Hefei	N/A	N/A	Haozhou road market	packaging in low temperature	Fresh
903		HFC864	caraway	Hefei	N/A	N/A	Zhong market	packaging in low temperature	Fresh
904		HFC865	caraway	Hefei	N/A	N/A	Century Lianhua Supermarket	packaging in low temperature	Fresh
905		HFJ881	cucumber	Hefei	N/A	N/A	Zhong market	normal temperature in bulk	Fresh
906		HFJ896	cucumber	Hefei	N/A	N/A	Xinghua market	normal temperature in bulk	Fresh
907		NCC913	lettuce	Nanchang	Nanchang	N/A	Wal-Mart	normal temperature in bulk	No water, no root
908		NCC914	caraway	Nanchang	Nanchang	N/A	RT-Mart	normal temperature in bulk	No water, no root
909		NCC915	tomato	Nanchang	Nanchang	N/A	Wal-Mart	normal temperature in bulk	No water
910		NCJ931	caraway	Nanchang	Nanchang	N/A	Dunzitang Fair	normal temperature in bulk	No water
911		NCN946	cucumber	Nanchang	Nanchang	N/A	Xi'mazhuang Fair	normal temperature in bulk	Fresh
912		WHC963	lettuce	Wuhan	Hubei	20121009	Lotus	normal temperature in bulk	No water, no root
913		WHC964	caraway	Wuhan	Hubei	20121009	Wal-Mart	normal temperature in bulk	No water, no root
914		WHC965	crown daisy	Wuhan	Hubei	20121009	Lotus	normal temperature in bulk	No water, no root
915		WHJ981	caraway	Wuhan	Wuhan	20121009	Tujialing Raw Fair	open-air in normal temperature	No water, no root
916		WHN996	cucumber	Wuhan	Wuhan	20121009	Yushun meat market	open-air in normal temperature	No water, no root
917		CDC1013	lettuce	Chengdu	Chengdu	20121016	Yankou people's shopping malls	normal temperature in bulk	fresh
918		CDC1014	caraway	Chengdu	Chengdu	20121016	Haolaiwu household mall	normal temperature in bulk	No water
919		CDC1015	tomato	Chengdu	Chengdu	20121016	Haolaiwu household mall	normal temperature in bulk	No water
920		CDJ1031	caraway	Chengdu	Chengdu	20121016	Bluestone bridge market	normal temperature in bulk	No water
921		CDN1046	cucumber	Chengdu	Chengdu	20121016	Xiaojiahe market	normal temperature in bulk	No water
922		KMC1063	lettuce	Kunming	Kunming	2012.11.04	Carrefour	normal temperature in bulk	N/A
923		KMC1064	caraway	Kunming	Kunming	2012.11.04	Carrefour	normal temperature in bulk	N/A
924		KMC1065	cucumber	Kunming	Kunming	2012.11.04	Wal-Mart	packaging in normal temperature	N/A
925		KMJ1081	caraway	Kunming	Kunming	2012.11.04	Tianyuanli yintan farmer's markets	normal temperature in bulk	N/A
926		KMN1096	radish	Kunming	Kunming	2012.11.04	Tianyuanli yintan farmer's markets	normal temperature in bulk	N/A
927		LZC1113	lettuce	Lanzhou	N/A	N/A	China Resources Vanguard	normal temperature in bulk	Fresh
928		LZC1114	cucumber	Lanzhou	N/A	N/A	Lanzhou Hualian Supermarket	packaging in normal temperature	Fresh
929		LZC1115	tomato	Lanzhou	N/A	N/A	China Resources Vanguard	normal temperature in bulk	Fresh
930		LZJ1131	caraway	Lanzhou	Lanzhou	N/A	Zhangye Road market	open-air in low temperature	Fresh
931		LZN1146	cucumber	Lanzhou	N/A	N/A	Bulan road street market	open-air in low temperature	Fresh
932		HEBC1163	lettuce	Haerbin	Haerbin	2012.11.20	Zhongyanghong supermarket	packaging in normal temperature	N/A
933		HEBC1164	cucumber	Haerbin	Haerbin	2012.11.20	Wal-Mart	normal temperature in bulk	N/A
934		HEBC1165	caraway	Haerbin	Haerbin	2012.11.20	Wal-Mart	packaging in normal temperature	N/A
935		HEBJ1181	caraway	Haerbin	Haerbin	2012.11.20	Hada market	normal temperature in bulk	N/A
936		HEBN1196	cucumber	Haerbin	Haerbin	2012.11.20	Daoli market	normal temperature in bulk	N/A
937		XAC1213	lettuce	Xi'an	Xi'an	2012.11.29	Wal-Mart	packaging in normal temperature	N/A

938		XAC1214	caraway	Xi'an	Xi'an	2012.11.29	China Resources Vanguard	normal temperature in bulk	N/A
939		XAC1215	cucumber	Xi'an	Xi'an	2012.11.29	Wal-Mart	normal temperature in bulk	N/A
940		XAJ1231	tomato	Xi'an	Xi'an	2012.11.29	Guanghuixiang Fair	normal temperature in bulk	N/A
941		XAN1246	onion	Xi'an	Xi'an	2012.11.29	Wangjiacun Fair	normal temperature in bulk	N/A
942		TYC1263	lettuce	Taiyuan	Taiyuan	20121211	Meitehao supermarket	normal temperature in bulk	N/A
943		TYC1264	caraway	Taiyuan	Taiyuan	20121211	Meitehao supermarket	normal temperature in bulk	N/A
944		TYC1265	cucumber	Taiyuan	Taiyuan	20121211	Wal-Mart	normal temperature in bulk	N/A
945		TYJ1281	caraway	Taiyuan	Taiyuan	20121211	Upwell Supermarket	normal temperature in bulk	N/A
946		TYN1296	cucumber	Taiyuan	Taiyuan	20121211	Upwell Supermarket	normal temperature in bulk	N/A
947		BJC1313	lettuce	Beijing	N/A	12.12.18	Carrefour	packaging in normal temperature	N/A
948		BJC1314	caraway	Beijing	N/A	12.12.18	Wal-Mart	normal temperature in bulk	N/A
949		BJC1315	cucumber	Beijing	N/A	12.12.18	Wal-Mart	packaging in normal temperature	N/A
950		BJJ1331	tomato	Beijing	N/A	12.12.18	Nongguangli Fair	normal temperature in bulk	N/A
951		BJN1346	eggplant	Beijing	N/A	N/A	Dingsheng market	normal temperature in bulk	N/A
952		JNC1363	lettuce	Jinan	N/A	N/A	TESCO	packaging in normal temperature	No water
953		JNC1364	caraway	Jinan	N/A	N/A	Wal-Mart	normal temperature in bulk	No water, no root
954		JNC1365	cucumber	Jinan	N/A	N/A	TESCO	packaging in normal temperature	Fresh
955		JNJ1381	caraway	Jinan	Jinan	20121224	Weiyi road Fair	cold storage in bulk	Fresh with root
956		JNN1396	lettuce	Jinan	N/A	N/A	Weiyi road Fair	cold storage in bulk	Fresh
957		YXC1413	lettuce	Guangzhou	N/A	N/A	Wal-market	packaging in normal temperature	No water, no root
958		YXC1414	caraway	Guangzhou	N/A	N/A	Tesco	normal temperature in bulk	No water, no root
959		YXC1415	cucumber	Guangzhou	N/A	N/A	Wal-market	packaging in normal temperature	No water, no root
960		YXJ1440	tomato	Guangzhou	N/A	N/A	Huifu new street Fair	normal temperature in bulk	No water, no root
961		YXJ1441	cucumber	Guangzhou	N/A	N/A	Shaheding market	normal temperature in bulk	N/A
962		LWC1463	lettuce	Guangzhou	Guangzhou	20130312	Carrefour	packaging in normal temperature	N/A
963		LWC1464	lettuce	Guangzhou	N/A	20130312	Carrefour	packaging in normal temperature	N/A
964		LWC1465	cucumber	Guangzhou	Guangzhou	20130312	Parknshop	packaging in normal temperature	N/A
965		LWN1490	tomato	Guangzhou	Guangzhou	20130312	Zilai meat market	normal temperature in bulk	N/A
966	**Vegetables**	LWN1491	lettuce	Guangzhou	Guangzhou	20130312	Longjin middle road Hongfu market	normal temperature in bulk	N/A
967		PYC1513	lettuce	Guangzhou	Guangzhou	20130319	China Resources Vanguard	normal temperature in bulk	N/A
968		PYC1514	cucumber	Guangzhou	Guangzhou	20130319	Parknshop	normal temperature in bulk	N/A
969		PYC1515	cabbage	Guangzhou	Guangzhou	20130319	China Resources Vanguard	normal temperature in bulk	N/A
970		PYN1540	tomato	Guangzhou	Guangzhou	20130319	Rainbow meat vegetable market	normal temperature in bulk	N/A
971		PYN1541	lettuce	Guangzhou	Guangzhou	20130319	Jushu meat vegetable market	normal temperature in bulk	N/A
972		CHC1563	lettuce	Guangzhou	N/A	N/A	Wanhe shopping mall	open-air in normal temperature	fresh
973		CHC1564	caraway	Guangzhou	N/A	N/A	Yonghenglong west street supermarket	open-air in normal temperature	fresh
974		CHC1565	cucumber	Guangzhou	N/A	N/A	Wanhe shopping mall	open-air in normal temperature	fresh
975		CHJ1590	tomato	Guangzhou	N/A	N/A	west street market	open-air in normal temperature	fresh
976		CHJ1591	cucumber	Guangzhou	N/A	N/A	Hedong meat vegetable market	open-air in normal temperature	fresh
977		ZCC1613	cabbage	Guangzhou	N/A	N/A	Renrenle Zengcheng shopping mall	packing in normal temperature	fresh
978		ZCC1614	caraway	Guangzhou	N/A	N/A	RT-Mart	open-air in normal temperature	fresh

979	ZCC1615	cucumber	Guangzhou	N/A	N/A	Renrenle Zengcheng shopping mall	packing in normal temperature	fresh
980	ZCJ1640	tomato	Guangzhou	N/A	N/A	Licheng east Fair	normal temperature in bulk	fresh
981	ZCJ1641	cucumber	Guangzhou	N/A	N/A	Xiajie Fair	normal temperature in bulk	fresh
982	SZC1663	tomato	Shenzhen	N/A	N/A	China Resources Vanguard	normal temperature in bulk	fresh
983	SZC1664	caraway	Shenzhen	N/A	N/A	Xinyijia	normal temperature in bulk	fresh
984	SZC1665	cabbage	Shenzhen	N/A	N/A	China Resources Vanguard	normal temperature in bulk	fresh
985	SZJ1690	tomato	Shenzhen	N/A	N/A	Mingli market	normal temperature in bulk	fresh
986	SZJ1691	lettuce	Shenzhen	N/A	N/A	Mingli market	normal temperature in bulk	fresh
987	STC1713	lettuce	Shantou	N/A	N/A	Lotus	normal temperature in bulk	fresh
988	STC1714	caraway	Shantou	N/A	N/A	Wal-Mart	normal temperature in bulk	fresh
989	STC1715	cucumber	Shantou	N/A	N/A	Wal-Mart	normal temperature in bulk	fresh
990	STJ1740	tomato	Shantou	N/A	N/A	Longkou north road market	normal temperature in bulk	fresh
991	STJ1741	lettuce	Shantou	N/A	N/A	Donghu street market	normal temperature in bulk	fresh
992	ZJC1763	tomato	zhangjiang	N/A	20130526	Wal-Mart	normal temperature in bulk	Fresh
993	ZJC1764	lettuce	zhangjiang	N/A	20130526	Aihua supermarket	normal temperature in bulk	Fresh
994	ZJC1765	cabbage	zhangjiang	N/A	20130526	Wal-Mart	normal temperature in bulk	Fresh
995	ZJJ1790	tomato	zhangjiang	N/A	20130526	Cikan Nanhua market	normal temperature in bulk	Fresh
996	ZJJ1791	lettuce	zhangjiang	N/A	20130526	Cikan north bridge market	normal temperature in bulk	Fresh
997	SGC1813	lettuce	Shaoguan	N/A	N/A	Wal-Mart	normal temperature in bulk	fresh
998	SGC1814	caraway	Shaoguan	N/A	N/A	RT-Mart	normal temperature in bulk	fresh
999	SGC1815	cucumber	Shaoguan	N/A	N/A	Wal-Mart	normal temperature in bulk	fresh
1000	SGJ1840	tomato	Shaoguan	N/A	N/A	Xinglong market	normal temperature in bulk	fresh
1001	SGJ1841	cucumber	Shaoguan	N/A	N/A	Fengcai market	normal temperature in bulk	fresh
1002	HYC1863	lettuce	Heyuan	N/A	N/A	Guangsheng supermarket	normal temperature in bulk	fresh
1003	HYC1864	caraway	Heyuan	N/A	N/A	Renrenle supermarket	normal temperature in bulk	fresh
1004	HYC1865	cucumber	Heyuan	N/A	N/A	Guangsheng supermarket	normal temperature in bulk	fresh
1005	HYJ1890	tomato	Heyuan	N/A	N/A	Yuancheng center market	normal temperature in bulk	fresh
1006	HYJ1891	cucumber	Heyuan	N/A	N/A	Xingyuan market	normal temperature in bulk	fresh
1007	FZC2213	tomato	Fuzhou	N/A	N/A	Carrefour	normal temperature in bulk	N/A
1008	FZC2214	lettuce	Fuzhou	N/A	N/A	Wal-Mart	normal temperature in bulk	N/A
1009	FZC2215	cucumber	Fuzhou	N/A	N/A	Carrefour	normal temperature in bulk	N/A
1010	FZJ2240	tomato	Fuzhou	N/A	N/A	Xiyingli market	normal temperature in bulk	Fresh
1011	FZJ2241	cucumber	Fuzhou	N/A	N/A	Xiyingli market	normal temperature in bulk	Fresh
1012	NNC2263	tomato	Nanning	N/A	N/A	China Resources Vanguard	normal temperature in bulk	Fresh
1013	NNC2264	caraway	Nanning	N/A	N/A	Wal-Mart	normal temperature in bulk	Fresh
1014	NNC2265	cucumber	Nanning	N/A	N/A	China Resources Vanguard	normal temperature in bulk	Fresh
1015	NNJ2290	tomato	Nanning	N/A	N/A	Dancun market	normal temperature in bulk	Fresh
1016	NNJ2291	cucumber	Nanning	N/A	N/A	Dancun market	normal temperature in bulk	Fresh
1017	XMC2313	tomato	Xiamen	N/A	N/A	Wal-Mart	open-air in normal temperature	fresh
1018	XMC2314	caraway	Xiamen	N/A	N/A	Tesco	open-air in normal temperature	fresh
1019	XMC2315	cucumber	Xiamen	N/A	N/A	Wal-Mart	open-air in normal temperature	fresh

1020		XMJ2340	tomato	Xiamen	N/A	N/A	The eight market	open-air in normal temperature	fresh
1021		XMJ2341	cucumber	Xiamen	N/A	N/A	Ruijing market	open-air in normal temperature	fresh
1022		BHC2363	tomato	Beihai	N/A	N/A	RT-Mart	open-air in normal temperature	fresh
1023		BHC2364	cucumber	Beihai	N/A	N/A	Hean supermarket	open-air in normal temperature	fresh
1024		BHC2365	cucumber	Beihai	N/A	N/A	RT-Mart	open-air in normal temperature	fresh
1025		BHJ2390	tomato	Beihai	N/A	N/A	Guizhou road market	open-air in normal temperature	fresh
1026		BHJ2391	cucumber	Beihai	N/A	N/A	Beijing road market	open-air in normal temperature	fresh
1027		HKC2413	tomato	Haikou	N/A	N/A	Parknshop	open-air in normal temperature	fresh
1028		HKC2414	tomato	Haikou	N/A	N/A	Carrefour	open-air in normal temperature	fresh
1029		HKC2415	cucumber	Haikou	N/A	N/A	Parknshop	open-air in normal temperature	fresh
1030		HKJ2440	tomato	Haikou	N/A	N/A	Longshepo market	open-air in normal temperature	fresh
1031		HKJ2441	cucumber	Haikou	N/A	N/A	Mingsheng market	open-air in normal temperature	fresh
1032		SYC2463	tomato	Sanya	N/A	N/A	Haowang supermarket	open-air in normal temperature	N/A
1033		SYC2464	caraway	Sanya	N/A	N/A	Fuleduo supermarket	open-air in normal temperature	N/A
1034		SYC2465	cucumber	Sanya	N/A	N/A	Haowang supermarket	open-air in normal temperature	N/A
1035		SYJ2490	lettuce	Sanya	N/A	N/A	The one market	open-air in normal temperature	N/A
1036		SYJ2491	cucumber	Sanya	N/A	N/A	City center market	open-air in normal temperature	N/A
